# Stroke and Alzheimer’s Disease: A Mendelian Randomization Study

**DOI:** 10.3389/fgene.2020.00581

**Published:** 2020-07-14

**Authors:** Tao Wang, Qing-bin Ni, Kun Wang, Zhifa Han, Bao-liang Sun

**Affiliations:** ^1^Academy for Advanced Interdisciplinary Studies, Peking University, Beijing, China; ^2^Postdoctoral Workstation, Taian City Central Hospital, Taian, China; ^3^School of Pharmaceutical Sciences, Tsinghua University, Beijing, China; ^4^Department of Neurology, The Second Affiliated Hospital, Shandong First Medical University & Shandong Academy of Medical Sciences, Taian, China; ^5^Key Laboratory of Cerebral Microcirculation in Universities of Shandong, Shandong First Medical University & Shandong Academy of Medical Sciences, Taian, China

**Keywords:** different kinds of stroke, Alzheimer’s disease, genome-wide association study, Mendelian randomization, pleiotropy

## Abstract

Stroke and Alzheimer’s disease (AD) are common neurological diseases. Several exiting studies indicated that late onset-AD and ischemic stroke have shared genetic links. Different kinds of stroke have different mechanisms. However, it remains unclear whether there is a causal relationship between different types of strokes, including any stroke (AS), any ischemic stroke (AIS), large-artery atherosclerotic stroke (LAS), and cardio-embolic stroke (CES), and AD. Herein, we conducted several Mendelian randomization (MR) studies to explore genetically causal link of different kinds of strokes and AD. The results for inverse-variance weighted (IVW) meta-analysis (β = −0.039, OR = 0.9618, and *P*-value = 0.750) and weighted median regression (WMR) (β = −0.156, OR = 0.8556, and *P*-value = 0.274) demonstrated that AS is not causally associated with AD risk. The result of MR-Egger regression (β = −1.312, *P*-value = 0.098) and intercept term (*P*-value = 0.105) illustrated no pleiotropy in this MR study. According to the results for IVW (*P*-value = 0.305, β = −0.103, and OR = 0.9021) and WMR (*P*-value = 0.487, β = −0.092, and OR = 0.9121) in the MR study between AIS and AD, there is no causal association between AIS and AD risk. In addition, the MR-Egger regression (*P*-value = 0.290 and β = −0.512) and intercept term (*P*-value = 0.387) showed no potential pleiotropy. LAS is not causally associated with AD risk according to the MR results (IVW: *P*-value = 0.568, β = 0.037, and OR = 1.0377; WMR: *P*-value = 0.793, β = −0.022, and OR = 0.9782). Additionally, the results of MR-Egger regression (*P*-value = 0.122 and β = −1.220) and intercept term (*P*-value = 0.110) showed no potential pleiotropy. Our results [IVW: *P*-value = 0.245, β = −0.064, and OR = 0.938; WMR: *P*-value = 0.331, β = −0.057, and OR = 0.9446; MR-Egger: *P*-value = 0.673 and β = −0.062, and intercept term (*P*-value = 0.985)] further demonstrated there is no causal link between CES and AD and no pleiotropy in this MR study. In conclusion, different types of stroke, including AS, AIS, LAS, and CES, would not be causally associated with AD risk.

## Introduction

Stroke is a kind of common neurological disease (Vijayan et al., 2017), and it is the second most common cause of death and disability all over the world (DALYs and Collaborators, 2016; Wang et al., 2016). Broadly, stroke can be classified into ischemic stroke and hemorrhagic stroke (Fatahzadeh and Glick, 2006). Ischemic stroke is caused by the infarction of the brain, which accounts for approximately 85% of all cases of stroke (Rosamond et al., 2008). Hemorrhagic stroke, comprising a small proportion (about 15%) of all strokes, is caused by brain bleed (Felberg and Naidech, 2003; Fatahzadeh and Glick, 2006; Vijayan and Reddy, 2016). There are several different subtypes of ischemic stroke according to the Trial of Org 10172 in Acute Stroke Treatment (TOAST) classification (Adams et al., 1993), including Large-vessel atherothrombosis, cardioembolism, small-vessel disease, other determining causes, and undetermined causes. Hemorrhagic stroke can be further classified into two subtypes, comprising subarachnoid hemorrhage (SAH) and intracerebral hemorrhage (ICH) (Felberg and Naidech, 2003). It is reported that around 90% of stroke survivors have different types of disability (Vercelli et al., 2017), which severely affects daily life for patients and results in high cost (Baumann et al., 2014). Most importantly, stroke is now considered a public health problem for society (Baumann et al., 2014).

Alzheimer’s disease (AD) is a degenerative neurological disease (Zhang et al., 2020) characterized by cognitive deterioration with loss of memory and behavioral changes (Katzman, 1986; Angelucci et al., 2010). The daily life activities for patients are affected by AD (Scheltens et al., 2016). The exiting evidence demonstrated that single nucleotide polymorphisms (SNPs) and nearby genes are significantly associated with AD (Lambert et al., 2009, 2013; Harold et al., 2013). The ε4 allele of the apolipoprotein E (*APOE*) gene is an important genetic risk factor for AD (Farrer et al., 1997). A rough estimate shows that genetic factors can lead to a 60–80% risk of AD (Gatz et al., 2006; Lambert et al., 2009; Sleegers et al., 2010).

An increasing number of studies have indicated that there are shared genetic links between AD and ischemic stroke. *APOE* is also a risk factor for several cerebrovascular diseases. In terms of pathological aspect, AD could lead to an increase in the risk of ischemic stroke (Chi et al., 2013; Tolppanen et al., 2013) and vice versa (Gamaldo et al., 2006). In addition, cerebrovascular events are related to a faster decline in AD patients (Regan et al., 2006). Moreover, tau protein, which is an important marker for AD, could exacerbate brain damage in a stroke animal model by mediating excitotoxic Ras/ERK signaling (Bi et al., 2017). Finally, several studies have shown that cerebrovascular disease is significantly associated with the deterioration of some clinical symptoms of AD (Snowdon et al., 1997).

Several existing genome-wide association studies (GWASs) have identified many SNPs associated with stroke (Traylor et al., 2012; Malik et al., 2018) and AD (Harold et al., 2009; Lambert et al., 2013). Accordingly, AD and ischemic stroke may share some genetic links. As we all know, ischemic stroke is only one type of stroke. The mechanisms of different kinds of stroke are distinct. It is still unclear whether there is a causal link between different types of stroke and AD. Mendelian randomization (MR) provides an approach for investigating the causal nature of several environmental exposures (Smith and Ebrahim, 2003). MR is a statistical method for causal inference from the observational study by using genetic variants (Verduijn et al., 2010; Davey Smith and Hemani, 2014). Recent studies have described the MR in detail (Davey Smith and Hemani, 2014; Emdin et al., 2017; Larsson et al., 2017). Here, we would like to analyze the causal association between different kinds of stroke, including any stroke (AS), any ischemic stroke (AIS), large-artery atherosclerotic stroke (LAS) and cardio-embolic stroke (CES), and AD by performing several MR studies.

## Materials and Methods

### Study Design

**FIGURE 1:**
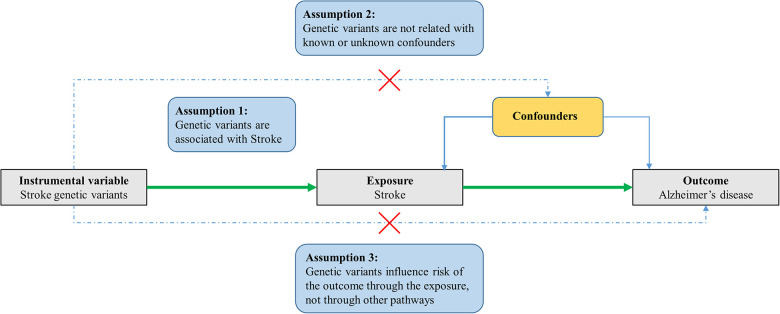
Three assumptions for MR analysis. Firstly, the genetic variants, which are selected as the instrumental variables, are associated with the exposure (stroke). Secondly, genetic variants are not related to known or unknown confounder factors. Thirdly, genetic variants would have an influence on the outcome (AD) only through exposure (stroke), not through other pathways.

In general, MR is based on three conditional assumptions (Figure 1). The first assumption is that the genetic variants, which are selected as instrumental variable, are associated with exposure (stroke). The second one is that genetic variants are not related to known or unknown confounder factors. The last assumption is that genetic variants would have an influence on the outcome (AD) only through exposure (stroke) and not through other pathways (Davey Smith and Hemani, 2014; Emdin et al., 2017; Larsson et al., 2017). This MR study was performed by using previously published, publicly available, large-scale GWAS summary datasets. All participants provided written informed consent in the corresponding original GWASs.

### Stroke GWAS Dataset

A stroke GWAS summary dataset was obtained from a large-scale multi-ancestry stroke genome-wide association meta-analysis, which includes 67,162 stroke cases and 454,450 normal controls (521,612 subjects in total) (Malik et al., 2018). Trans-ancestral and ancestry-specific meta-analyses were conducted for AS, AIS, and common subtypes of stroke, including LAS, CES, and small-vessel stroke (SVS) (Malik et al., 2018). A total of 32 significant SNPs, including 22 novel SNPs, were identified from the study (Malik et al., 2018). 12 SNPs for AS phenotype among which were obtained by trans-ancestral meta-analysis (Malik et al., 2018). Nine SNPs for the AIS phenotype were identified by trans-ancestral meta-analysis, and only two SNPs were identified for the AIS phenotype using ancestry-specific meta-analysis (Malik et al., 2018). Three SNPs and two SNPs for the LAS phenotype were obtained by trans-ancestral meta-analysis and ancestry-specific meta-analysis, respectively (Malik et al., 2018). Three SNPs for the CES phenotype were obtained by trans-ancestral meta-analysis, and only one SNP was identified for CES by ancestry-specific meta-analysis (Malik et al., 2018). AS is comprised of all types of stroke. Twelve SNPs associated with AS were used for further analysis in this article. The rs12476527 was removed because *P*-value in the GWAS summary dataset is 6.44E–8 (>5E-8). Therefore, the remaining 11 SNPs were used as the instrumental variables for MR analysis between AS and AD. The AS GWAS summary dataset was downloaded from GWAS Catalog (accession ID: GCST005838). As for 11 SNPs for the AIS phenotype, five SNPs for the LAS phenotype, and four SNPs for the CES phenotype, we can obtain their summary information, including the beta, standard error, and *P*-value, from a published article (Malik et al., 2018). Beta can be calculated by the formula: Beta = *ln*⁡(OR), and standard error (SE) can be obtained according to the formula:

$$\text{SE}=\frac{\left(\ln\left(C\,I_{\text{upper}}\right)-\ln\left(C\,I_{\text{lower}}\right)\right)}{2\times 1.96},$$

where *CI*_*upper*_ and *CI*_*lower*_ refer to the upper confidence interval and the lower confidence interval of OR, respectively. The age information for each study was obtained from the published article (Malik et al., 2018), which is shown in Supplementary Table 1. There are 60,742 stroke cases excluding COMPASS, RACE2, and SLESS studies. The average age for 60,742 stroke cases is approximately 68.5 years old.

### AD GWAS Dataset

The AD GWAS summary data was downloaded from the International Genomics of Alzheimer’s Project (IGAP), which conducted a large-scale meta-analysis in 2013 (Lambert et al., 2013). IGAP is actually a two-stage study on the subjects of European descent (Lambert et al., 2013). Briefly, in stage 1, IGAP firstly genotyped and imputed 7,055,881 SNPs, and then conducted a meta-analysis on four GWAS summary data sets from four consortia (ADGC, CHARGE, EADI, and GERAD), which were comprised of 17,008 AD patients and 37,154 normal controls (Lambert et al., 2013). The age information for each consortium was provided in Supplementary Table 2. The mean age at onset for 17,008 AD cases is about 74.18 years old. In stage 2, 11,632 SNPs were used to test the association in another dataset, which includes 8,572 AD patients and 11,312 normal controls (Lambert et al., 2013). All AD cases met the criteria for the National Institute of Neurological and Communicative Disorders and Stroke (NINCDS), the Alzheimer’s Disease and Related Disorders Association (ADRDA), or guidelines for Diagnosis and statistical Manual of Mental Disorders (DSMMD) (Lambert et al., 2013). In this article, the stage 1 GWAS dataset was used to extract the summary statistics, including *P*-value, odds ratio, and standard error, of the 11 genetic variants of the AS phenotype, 11 genetic variants for the AIS phenotype, five genetic variants for the LAS phenotype, and four genetic variants for the CES phenotype.

### Pleiotropy Analysis

When we perform an MR study, there will be potential violation of assumptions two and three (Figure 1). Specifically, when the genetic instrument variable is related to the outcome not only through exposure but also through other biological pathways. Therefore, it is important to assess the pleiotropy in an MR study, which can ensure that the selected genetic variants (instrumental variable) will not have an effect on the outcome through other biological pathways except for exposure (stroke).

Recently, nine potential risk factors for dementia were identified in a review, which comprises low levels of education, midlife hearing loss, physical inactivity, hypertension, type 2 diabetes, obesity, smoking, depression, and social isolation (Livingston et al., 2017). In stage 1, we will consider these nine risk factors to assess the pleiotropy. In addition, alcohol associated with AD risk was identified (Livingston et al., 2017). In stage 2, we will evaluate the association between instrumental variable and alcohol. The three AD biomarkers, including cerebrospinal fluid tau, tau phosphorylated at threonine 181 (ptau), and amyloid-beta1–42 (Aβ42), were described (Deming et al., 2017). In stage 3, the association between the selected genetic variants (instrumental variable) and these three AD biomarkers was investigated. In stage 4, a statistical method called the MR-Egger test was used to assess the potential pleiotropy association between the selected genetic variants (instrumental variable) and potential confounder factors (Dale et al., 2017; Tillmann et al., 2017). A systematic review reported that telomeres length, smoking quantity, vitamin D, homocysteine, systolic blood pressure, fasting glucose, insulin sensitivity, and high-density lipoprotein cholesterol are associated with dementia risk (Kuzma et al., 2018). In stage 5, we further assessed the association between the instrumental variable and these risk factors for dementia. In stage 1–3 and 5, we used the *P*-value < 0.004545 (0.05/11) as the significant cutoff for association after Bonferroni correction for AS stroke.

### MR Methods

In this study, we performed an MR analysis based on two statistic methods, including inverse-variance weighted (IVW) meta-analysis and weighted median regression (WMR), which have been described well in the previous studies (Davey Smith and Hemani, 2014; Dale et al., 2017; Emdin et al., 2017; Tillmann et al., 2017). At the same time, we used MR-Egger regression to evaluate the potential pleiotropy, which can be utilized to test for bias from pleiotropy (Bowden et al., 2015). The related MR analysis would be conducted by using R package MR (Yavorska and Burgess, 2017). As for the MR between AS and AD, 11 SNPs were used as the instrumental variable. As for the 11 SNPs for AIS, the summary information of rs34311906 cannot be obtained from the AD GWAS dataset. Additionally, the *P*-value of rs42039 in the AD GWAS dataset is less than 0.05. Therefore, we used nine SNPs as the instrumental variable in the MR between AIS stroke and AD. Three SNPs, after removing rs7610618 and rs17612742, were used as the instrumental variable in the MR study between LAS and AD. As for the MR between CES and AD, the instrumental variable includes three SNPs after removing rs146390073. The *P*-value < 0.05 was considered a significant statistical threshold in this article.

## Results

### Instrumental Selection for AS (Genetic Variants Significantly Associated With AS)

We obtained 11 genetic variants as the instrumental variable according to their GWAS *P*-value < 5E-8, which are rs880315, rs12037987, rs16896398, rs7859727, rs2295786, rs35436, rs9526212, rs8103309, rs1052053, rs4959130, and rs12445022. These 11 genetic variants were identified for AS by trans-ancestral meta-analysis. These genetic variants are located in different genes. Therefore, there is no linkage disequilibrium among these genetic variants. Detailed information of 11 genetic variants is shown in Table 1.

**TABLE 1:** Characteristics of 11 genetic variants in AS and AD GWAS datasets.

SNP	Chr	Nearby genes	EA^a^	NEA	EAF^b^	AS GWAS			AD GWAS		
						Beta^c^	SE^c^	*P*-value^c^	Beta^d^	SE^d^	*P*-value^d^
rs880315	1	*CASZ1*	C	T	0.4	0.0527	0.0084	3.619E-10	–0.0083	0.0171	0.6301
rs12037987	1	*WNT2B*	C	T	0.16	0.071	0.0128	2.734E-08	–0.0058	0.0333	0.8606
rs16896398	6	*SLC22A7-ZNF318*	T	A	0.34	0.0477	0.0084	1.301E-08	–0.0072	0.017	0.6719
rs7859727	9	*Chr9p21*	T	C	0.53	0.0494	0.0079	4.221E-10	0.024	0.0156	0.1244
rs2295786	10	*SH3PXD2A*	A	T	0.6	0.0526	0.0082	1.797E-10	–0.0263	0.0162	0.1043
rs35436	12	*TBX3*	C	T	0.62	0.0462	0.0083	2.865E-08	0.0112	0.0165	0.4949
rs9526212	13	*LRCH1*	G	A	0.76	0.0587	0.0094	5.034E-10	–0.0187	0.0181	0.3015
rs8103309	19	*SMARCA4-LDLR*	T	C	0.65	0.0501	0.0091	3.397E-08	0.0249	0.0172	0.1487
rs1052053	1	*PMF1- SEMA4A*	G	A	0.4	0.0624	0.0082	2.699E-14	–0.0209	0.016	0.1898
rs4959130	6	*FOXF2*	A	G	0.14	0.0779	0.0129	1.424E-09	–0.033	0.0236	0.1625
rs12445022	16	*ZCCHC14*	A	G	0.31	0.0574	0.0089	1.048E-10	0.0303	0.0167	0.07066

*SNP, single-nucleotide polymorphism; Chr, chromosome; EA, effect allele; NEA, non-effect allele; EAF, effect allele frequency; AS, any stroke; AD, Alzheimer’s disease; GWAS, genome-wide association studies; SE, standard error. ^*a*^Effect allele associated with stroke. ^*b*^The frequency of effect allele associated with stroke. ^*c*^The summary statistics for SNP, including Beta, standard error, and *P*-value, were obtained from any stroke GWAS dataset. Beta > 0 indicates this effect allele increases the stroke risk. Otherwise, it reduces the stroke risk (Beta < 0). ^*d*^The summary statistics for SNP, including Beta, standard error, and *P*-value, were obtained from AD GWAS dataset. The Beta was obtained based on the effect allele associated stroke. Beta > 0 indicates this effect allele increases the AD risk. Otherwise, it reduces the AD risk (Beta < 0).*

### Instrumental Selection for AIS (Genetic Variants Significantly Associated With AIS)

As for the 11 SNPs for AIS, the summary information of rs34311906 and its proxy SNP cannot be obtained from the AD GWAS dataset. Because we did not find the summary information of rs6825454 in the AD GWAS dataset, we used the information of its proxy SNP rs56010410. Additionally, the *P*-value of rs42039 in the AD GWAS dataset is less than 0.05. Therefore, we used nine SNPs (rs6825454, rs11957829, rs7304841, rs4932370, rs11867415, rs2229383, rs635634, rs2005108, and rs3184504) as the instrumental variable in MR analysis between AIS and AD. A detailed description of these 11 SNPs for AIS is shown in Table 2.

**TABLE 2:** Characteristics of 11 genetic variants in AIS and AD GWAS datasets.

SNP	Chr	Nearby genes	EA^a^	NEA	EAF^b^	AIS GWAS			AD GWAS		
						Beta^c^	SE^c^	*P*-value^c^	Beta^d^	SE^d^	*P*-value^d^
rs34311906	4	*ANK2*	C	T	0.41	0.0677	0.0120	1.07E-08	NA	NA	NA
rs6825454	4	*FGA*	C	T	0.31	0.0583	0.0096	7.43E-10	0.0047^e^	0.0184^e^	0.7961^e^
rs11957829	5	*LOC100505841*	A	G	0.82	0.0677	0.0119	7.51E-09	0.0093	0.022	0.6732
rs42039	7	*CDK6*	C	T	0.77	0.0677	0.0120	6.55E-09	0.039	0.0187	0.03639
rs7304841	12	*PDE3A*	A	C	0.59	0.0488	0.0097	4.93E-08	−0.0142	0.0176	0.4183
rs4932370	15	*FURIN–FES*	A	G	0.33	0.0488	0.0097	2.88E-08	−0.0071	0.0171	0.6793
rs11867415	17	*PRPF8*	G	A	0.18	0.0862	0.0163	4.81E-08	−0.0392	0.0347	0.258
rs2229383	19	*ILF3–SLC44A2*	T	G	0.65	0.0488	0.0097	4.72E-08	0.0158	0.0169	0.3508
rs635634	9	*ABO*	T	C	0.19	0.0770	0.0142	9.18E-09	−0.0055	0.0195	0.7793
rs2005108	11	*MMP12*	T	C	0.12	0.0770	0.0142	3.33E-08	−0.0003	0.0231	0.9888
rs3184504	12	*SH2B3*	T	C	0.45	0.0770	0.0094	2.17E-14	−0.0252	0.0159	0.1134

*^*a*^Effect allele associated with AIS. ^*b*^The frequency of effect allele associated with AIS. ^*c*^The summary statistics for SNP, including Beta, standard error, and *P*-value, were obtained from the published article (Malik et al., 2018). ^*d*^The summary statistics for SNP, including Beta, standard error, and *P*-value, were obtained from AD GWAS dataset. ^*e*^The summary information of the proxy SNP rs56010410 for rs6825454 (*r*^2^ = 0.97), and the effect allele of rs56010410 is C.*

### Instrumental Selection for LAS (Genetic Variants Significantly Associated With LAS)

There are five genetic variants associated with LAS. The summary information of these SNPs is shown in Table 3. We did not find the summary information of rs7610618 and its proxy SNP from the AD GWAS dataset. The *P*-value of rs17612742 in AD GWAS dataset is 0.01618 (<0.05). Therefore, the remained 3 SNPs (rs10820405, rs12124533, rs2107595) after removing rs7610618 and rs17612742 were used as the instrumental variable in the MR between LAS and AD.

**TABLE 3:** Characteristics of five genetic variants in LAS and AD GWAS datasets.

SNP	Chr	Nearby genes	EA^a^	NEA	EAF^b^	LAS GWAS			AD GWAS		
						Beta^c^	SE^c^	*P*-value^c^	Beta^d^	SE^d^	*P*-value^d^
rs7610618	3	*TM4SF4–TM4SF1*	T	C	0.01	0.8459	0.1490	1.44E-8	NA	NA	NA
rs17612742	4	*EDNRA*	C	T	0.21	0.1740	0.0278	1.46E-11	−0.0562	0.0234	0.01618
rs10820405	9	*LINC01492*	G	A	0.82	0.1823	0.0341	4.51E-08	−0.0086	0.0198	0.6644
rs12124533	1	*TSPAN2*	T	C	0.24	0.1570	0.0262	1.22E-8	0.0321	0.0187	0.08658
rs2107595	7	*HDAC9–TWIST1*	A	G	0.24	0.1906	0.0233	3.65E-15	−0.0038	0.0211	0.8582

*^*a*^Effect allele associated with LAS. ^*b*^The frequency of effect allele associated with LAS. ^*c*^The summary statistics for SNP, including Beta, standard error, and *P*-value, were obtained from the published article (Malik et al., 2018). ^*d*^The summary statistics for SNP, including Beta, standard error, and *P*-value, were obtained from AD GWAS dataset.*

### Instrumental Selection for CES (Genetic Variants Significantly Associated With CES)

There are four SNPs that are associated with CES. The detailed information on these four SNPs is shown in Table 4. There are no summary statistics of rs146390073 and its proxy SNPs in the AD GWAS dataset. Thus, we used three SNPs (rs6891174, rs13143308, and rs12932445) as the instrumental variable in the MR between CES stroke and AD.

**TABLE 4:** Characteristics of four genetic variants in CES and AD GWAS datasets.

SNP	Chr	Nearby genes	EA^a^	NEA	EAF^b^	CES GWAS			AD GWAS		
						Beta^c^	SE^c^	*P*-value^c^	Beta^d^	SE^d^	*P*-value^d^
rs146390073	1	*RGS7*	T	C	0.02	0.6678	0.1205	2.20E-08	NA	NA	NA
rs6891174	5	*NKX2-5*	A	G	0.35	0.1044	0.0206	5.82E-09	−0.0021	0.017	0.9006
rs13143308	4	*PITX2*	T	G	0.34	0.2776	0.0193	1.86E-47	−0.0128	0.0188	0.4972
rs12932445	16	*ZFHX3*	C	T	0.21	0.1823	0.0213	6.86E-18	−0.0261	0.0216	0.2272

*^*a*^Effect allele associated with CES. ^*b*^The frequency of effect allele associated with CES. ^*c*^The summary statistics for SNP, including Beta, standard error, and *P*-value, were obtained from the published article (Malik et al., 2018). ^*d*^The summary statistics for SNP, including Beta, standard error, and *P*-value, were obtained from AD GWAS dataset.*

### Association of AS Genetic Variants With AD

These 11 genetic variants are significantly associated with AS. The summary statistics of these 11 genetic variants, including beta, standard error, and *P*-value, were also obtained from AD GWAS summary dataset (Table 1). The results showed that none of the 11 genetic variants was significantly related to the AD risk with *P*-value cutoff is 0.05. It can demonstrate that these 11 genetic variants are not directly associated with AD risk.

### Association of AIS, LAS, and CES Genetic Variants With AD

The summary statistics of 11 SNPs for AIS, five SNPs for LAS, and four SNPs for CES were calculated according to the data from the published article (Malik et al., 2018). According to the *P*-value cutoff of 0.05, rs42039 is associated with the AD risk, and nine SNPs are not associated with AD risk (Table 2). The three SNPs (rs10820405, rs12124533, and rs2107595) are not directly associated with AD risk (Table 3). Among the four SNPs for CES stroke, three SNPs (rs6891174, rs13143308, and rs12932445) are not associated with AD risk (Table 4).

### Pleiotropy Analysis

In stage 1, based on the PhenoScanner database (Staley et al., 2016; Kamat et al., 2019), the results for associations between all 11 genetic variants with nine potential risk factors for dementia are shown in Supplementary Table 3. According to significant cutoff (*P*-value < 0.004545), rs16896398 is associated with a midlife hearing loss phenotype (hearing difficulty or problems with background noise), a physical inactivity phenotype (number of days or week of vigorous physical activity 10+ min), and a type II diabetes phenotype. The rs880315 and rs8103309 are linked to hypertension and body mass index, respectively. There is the association between rs7859727 and rs9526212 and the type II diabetes phenotype. In stage 2, all 11 genetic variants were also not associated with alcohol because of their *P*-value (>0.004545) (Table 5). In stage 3, none of 11 genetic variants were significantly related with the three AD markers, including cerebrospinal fluid tau, tau phosphorylated at threonine 181 (ptau), and Aβ42 (Table 5). In stage 4, these 11 genetic variants did not show the pleiotropy in this MR study according to MR-Egger regression result (*P*-value = 0.098 and β = −1.312) and intercept term (*P*-value = 0.105). In stage 5, the association information between instrumental variable and other risk factors for dementia can be also obtained by the PhenoScanner database (Staley et al., 2016; Kamat et al., 2019), which can be accessed in Supplementary Table 4.

**TABLE 5:** *P*-values for 11 genetic variants with three AD marks (tauB, ptauB, Aβ42B) and alcohol phenotype.

SNP	tauB	ptauB	Aβ42B	Continuous alcohol	Dichotomous alcohol
	*P*-value	*P*-value	*P*-value	*P*-value	*P*-value
rs880315	0.652	0.9521	0.2221	0.6342	0.05835
rs12037987	0.8547	0.6817	0.03467	NA	NA
rs16896398	0.029	0.1406	0.0105	0.4732	0.7572
rs7859727	0.4139	0.1943	0.05511	0.4999	0.9692
rs2295786	0.05925	0.2305	0.5089	0.966	0.4138
rs35436	0.514	0.7463	0.3127	NA	NA
rs9526212	0.6056	0.5681	0.8516	NA	NA
rs1052053	0.2341	0.4901	0.6899	0.8213	0.1064
rs4959130	0.8545	0.6134	0.4263	NA	NA
rs12445022	0.8024	0.6333	0.08774	0.1053	0.1229
rs8103309	NA	NA	NA	NA	NA

### MR Analysis Between AS and AD Risk

We used the 11 genetic variants as the instrumental variables with which to perform the MR analysis between AS and AD risk. The results for IVW method (*P*-value = 0.750, β = −0.039, and OR = 0.9618) and WMR (*P*-value = 0.274, β = −0.156, and OR = 0.8556) indicate there is no causal association between AS stroke and AD risk. In addition, there is no potential pleiotropy for these 11 genetic variants according to the MR-Egger regression result (β = −1.312, *P*-value = 0.098) and intercept term (β = 0.071, *P*-value = 0.105). We also did not find the pleiotropy of 11 SNPs from the heterogeneity test statistic = 15.8637 on 10 degrees of freedom (*P*-value = 0.1036).

Moreover, we further conducted an MR analysis between AS and AD using the Steiger filtering method by R package TwoSampleMR. Steiger filtering was used to assess the directional test of instrumental variables. The rs16896398 and rs2295786 were removed for being palindromic with intermediate allele frequencies. The remaining nine SNPs were used for MR analysis. IVW result (*P*-value = 0.8492, β = 0.0259) and WMR result (*P*-value = 0.3956, β = −0.129) indicated that AS is not causally linked to AD risk. According to the MR Egger result (*P*-value = 0.0482, β = −1.725), there is a very weak association between AS and AD. The R script for this MR analysis can be obtained in Supplementary File 1.

### MR Analysis Between AIS and AD Risk

Nine SNPs were used to conduct the MR analysis between AIS and AD risk. The results for the IVW method are *P*-value = 0.305, β = −0.103, and OR = 0.9021, and the results for the WMR method are *P*-value = 0.487, β = −0.092, and OR = 0.9121, which demonstrated that there is no causal association between AIS and AD risk. The MR-Egger result (*P*-value = 0.290 and β = −0.512) and intercept term (*P*-value = 0.387 and β = 0.027) can show that there is no potential pleiotropy of these nine SNPs. In addition, according to heterogeneity test statistic = 4.7585 on 8 degrees of freedom (*P*-value = 0.7831), there is no pleiotropy of the nine SNPs in this MR analysis.

We also performed the MR analysis between AIS and AD using the Steiger filtering method. The nine SNPs associated with AIS were used as an instrumental variable for this MR study. According to the IVW result (*P*-value = 0.3053, β = −0.1025), WMR result (*P*-value = 0.4919, β = −0.0886) and MR Egger result (*P*-value = 0.3255, β = −0.5124), there is no causal association between AIS and AD risk. The R script for these two MR analyses can be obtained in Supplementary File 2.

### MR Analysis Between LAS and AD Risk

In this MR analysis, we used three SNPs as instrumental variables. The results for IVW method and WMR method are *P*-value = 0.568, β = 0.037, and OR = 1.0377 and *P*-value = 0.793, β = −0.022, and OR = 0.9782, respectively. According to these results, LAS is not causally linked to AD risk. In addition, there is no potential pleiotropy for these three SNPs according to the result of the MR-Egger method (*P*-value = 0.122 and β = −1.220) and intercept term (*P*-value = 0.110 and β = 0.222). Moreover, heterogeneity test statistic = 2.8418 on 2 degrees of freedom (*P*-value = 0.2415) shows that there is not pleiotropy of the instrumental variable in this analysis. Lastly, we also conducted single SNP MR analysis. When the only one SNP rs10820405 was used as an instrumental variable in MR analysis, the result for IVW method is *P*-value = 0.664 and β = −0.047. We used the only one SNP rs12124533 as instrumental variable, the IVW result for this MR analysis is *P*-value = 0.086 and β = 0.204. Additionally, the IVW result (*P*-value = 0.857 and β = −0.020) was obtained for SNP rs2107595 as an instrumental variable in the MR analysis.

The MR study between LAS and AD using the Steiger filtering method was conducted by the R package TwoSampleMR. The three SNPs were used as an instrumental variable in this MR study. According to the IVW result (*P*-value = 0.6320, β = 0.0371), WMR result (*P*-value = 0.7907, β = −0.0231) and MR Egger result (*P*-value = 0.3652, β = −1.2205), AIS is not causal linked to AD risk. The R script for these MR analyses can be obtained in Supplementary File 3.

### MR Analysis Between CES and AD Risk

The MR analysis was conducted by using three SNPs (rs6891174, rs13143308, and rs12932445). We found that there is no causal link between CES and AD risk according the results of IVW method (*P*-value = 0.245, β = −0.064, and OR = 0.938) and WMR method (*P*-value = 0.331, β = −0.057, and OR = 0.9446). Moreover, the potential pleiotropy for these 3 SNPs did not be observed in MR analysis because of the results of the MR-Egger method (*P*-value = 0.673 and β = −0.062) and intercept term (*P*-value = 0.985 and β = −0.001). In addition, the heterogeneity test statistic is 0.5889 on 2 degrees of freedom (*P*-value = 0.7450), which indicates there is not pleiotropy for the instrumental variable in this MR analysis. Finally, a single SNP MR analysis was also performed. We obtained the IVW result (*P*-value = 0.902 and β = −0.020) for single SNP rs6891174, the VW result (*P*-value = 0.496 and β = −0.046) for single SNP rs13143308, and the VW result (*P*-value = 0.227 and β = −0.143) for single SNP rs12932445 as instrumental variables in the MR analysis.

We further conducted the MR analysis between CES and AD using the Steiger filtering method. The three SNPs associated with CES were used as an instrumental variable in this MR study. We found there is no causal association between CES and AD according to the IVW result (*P*-value = 0.2453, β = −0.0643), WMR result (*P*-value = 0.3374, β = −0.05697) and MR Egger result (*P*-value = 0.7457, β = −0.0618). The R script for these MR analyses can be obtained in Supplementary File 4.

### MR Analysis Between AD and AS

In the published AD GWAS study (Lambert et al., 2013), there are 14 SNPs reaching genome-wide significance (rs6656401, rs6733839, rs10948363, rs9271192, rs11771145, rs28834970, rs9331896, rs983392, rs10792832, rs11218343, rs10498633, rs8093731, rs4147929, and rs3865444). They are not located in the *APOE* gene. We only obtained the summary statistics for 13 SNPs excepting for rs9271192 from the AD dataset, and the beta and standard error for rs9271192 were calculated from the OR and 95% confidence interval provided in the published article. The corresponding summary information for 13 SNPs excepting rs9271192 was obtained from AS dataset. The summary statistics for proxy SNP rs9271162 (*r*^2^ = 0.96) were used for rs9271192. Characteristics of fourteen genetic variants in AD and AS datasets are shown in Supplementary Table 5. Twelve SNPs were utilized as instrument variables in the MR analysis between AD and AS because of *P*-values of rs983392 and rs10792832 in AS dataset <0.05. Both of IVW result (*P*-value = 0.708, β = −0.007) and WMR result (*P*-value = 0.604, β = −0.013) indicate AD would not be causally associated with stroke risk. In addition, the MR-Egger regression (*P*-value = 0.639 and β = −0.019) and intercept term (*P*-value = 0.738 and β = 0.002) demonstrate there is no significant pleiotropy in the MR study between AD and AS.

We further used these 12 SNPs to perform another MR analysis using the Steiger filtering method by way of an R package TwoSampleMR. On the ground of the IVW result (*P*-value = 0.7079, β = −0.0070), WMR result (*P*-value = 0.5998, β = −0.0133), and MR-Egger result (*P*-value = 0.6491, β = −0.0190), we did not observe the causal association between AD and AS either. The R script for these two MR analyses can be obtained in Supplementary File 5.

### Sensitive Analysis for MR Study Between AS and AD

To assess the stability for MR analysis results between AS and AD, we further conducted the sensitive analyses using a leave-one-out method. In brief, the SNPs were excluded one by one for MR analysis. The results for sensitive analysis are shown in Table 6. For IVW analysis, although the direction of the genetic estimates between stroke and AD risk changed when excluding rs2295786, rs1052053, and rs4959130, the direction of genetic estimates remained constant largely when excluding other eight genetic variants (rs880315, rs12037987, rs16896398, rs7859727, rs35436, rs9526212, rs8103309, and rs12445022). Most importantly, the *P*-values for all IVW analysis is more than 0.05, which manifests no significant association between stroke and AD. For WMR, the direction of the genetic variants between stroke and AD remained unchanged due to all the β values < 0. In addition, the results for WMR showed there was no significant association between stroke and AD risk. For MR-Egger regression, although there is a little potential pleiotropy when excluding the rs2295786 (MR-Egger intercept term: *P*-value = 0.041) and rs12445022 (MR-Egger intercept term: *P*-value = 0.042), the results largely indicated no potential pleiotropy of the genetic variants when excluding the other nine genetic variants.

**TABLE 6:** The results for sensitive analysis of the selected 11 genetic variants.

SNP^a^	IVW		WMR		MR-Egger regression		MR-Egger intercept term	
	Beta	*P*-value	Beta	*P*-value	Beta	*P*-value	Beta	*P*-value
rs880315	−0.027	0.838	−0.192	0.211	−1.349	0.105	0.074	0.108
rs12037987	−0.037	0.778	−0.167	0.259	−1.440	0.103	0.078	0.109
rs16896398	−0.030	0.822	−0.193	0.197	−1.527	0.070	0.085	0.073
rs7859727	−0.093	0.443	−0.211	0.147	−1.097	0.177	0.057	0.212
rs2295786	0.011	0.926	−0.152	0.312	−1.432	0.046	0.081	0.041
rs35436	−0.061	0.640	−0.193	0.196	−1.299	0.152	0.070	0.168
rs9526212	−0.008	0.949	−0.152	0.325	−1.232	0.139	0.068	0.138
rs8103309	−0.085	0.487	−0.198	0.175	−1.150	0.153	0.060	0.181
rs1052053	0.010	0.939	−0.144	0.357	−1.133	0.197	0.063	0.189
rs4959130	0.005	0.971	−0.152	0.319	−1.240	0.283	0.067	0.279
rs12445022	−0.109	0.348	−0.225	0.119	−1.485	0.030	0.077	0.042

*^*a*^Excluding this SNP for analysis.*

### Sensitive Analysis for MR Study Between AD and AS

In order to evaluate the robustness of MR results for AD and AS, sensitive analysis for MR study between AD and AS was also performed using the leave-one-out method. The sensitive analysis results were shown in Table 7. According to the sensitive analysis results (*P*-values of IVW, WMR, and MR-Egger regression > 0.05), AD is not causally linked to AS. In addition, the *P*-values of the MR-Egger intercept term (>0.05) indicated there is no potential pleiotropy when excluding any SNP. The R script for sensitive analysis using leave-one-out approach can be obtained in Supplementary File 5.

**TABLE 7:** The sensitive analysis results of the 12 genetic variants.

SNP^a^	IVW		WMR		MR-Egger regression		MR-Egger intercept term	
	Beta	*P*-value	Beta	*P*-value	Beta	*P*-value	Beta	*P*-value
rs6656401	−0.011	0.578	−0.014	0.593	−0.021	0.607	0.002	0.770
rs6733839	−0.001	0.978	−0.010	0.725	−0.011	0.801	0.002	0.784
rs10948363	−0.009	0.652	−0.014	0.594	−0.017	0.700	0.001	0.836
rs9271192	−0.007	0.700	−0.014	0.594	−0.019	0.663	0.002	0.766
rs11771145	0.001	0.952	−0.013	0.607	−0.033	0.421	0.006	0.344
rs28834970	−0.007	0.734	−0.014	0.597	−0.021	0.633	0.003	0.713
rs9331896	−0.008	0.703	−0.014	0.601	−0.019	0.654	0.002	0.758
rs11218343	−0.005	0.803	−0.011	0.673	−0.016	0.735	0.002	0.796
rs10498633	−0.004	0.840	−0.013	0.607	−0.024	0.561	0.004	0.580
rs8093731	−0.005	0.811	−0.016	0.590	−0.026	0.723	0.003	0.766
rs4147929	−0.015	0.436	−0.014	0.588	−0.019	0.632	0.001	0.907
rs3865444	−0.013	0.491	−0.014	0.590	−0.007	0.860	−0.001	0.868

*^*a*^Excluding this SNP for analysis.*

## Discussion

Stroke and AD are common among neurological disease (Vijayan et al., 2017; Liu et al., 2018). To date, several exiting evidence manifested that AD and ischemic stroke may have shared genetic links (Gamaldo et al., 2006; Chi et al., 2013; Wei et al., 2019). However, it remains not clear whether different types of strokes are genetically associated with AD. Human genetic variants generally would be randomly assigned in the population (Emdin et al., 2017; Cornish et al., 2019). Therefore, they are not largely linked with the confounders and could be used as proxies for an exposure (Emdin et al., 2017; Cornish et al., 2019), which guarantees that MR could be less likely to be biased in several observational epidemiological studies and get the better of the methodological restrictions of randomized controlled studies (Davey Smith and Hemani, 2014; Liu et al., 2018; Cornish et al., 2019).

In this study, we performed several MR analyses to investigate the causal relationship between different kinds of stroke and AD. The result is that AS is not causally associated with AD (*P*-value = 0.750 and β = −0.039 for IVW method; *P*-value = 0.274 and β = −0.156 for WMR method). In addition, there is no causal link between other types of stroke, such as AIS, LAS, and CES, and AD according to our results, which means that AS would be not significantly associated with the development of AD. The average age of cases in stroke GWAS is about 68.5 years old, and the mean age at onset for 17,008 cases in AD GWAS is approximately 74.18 years old, which provides an overall idea of stroke would happen first in general. In addition, we also performed the reverse MR with AD as exposure and AS as an outcome. Our research results indicated AD is not causally linked to AS risk. Perhaps, stroke and AD are the two complication diseases, and their development and progression would be caused by other factors.

There are several advantages of our several MR analyses in this article. Firstly, we used the large-scale stroke GWAS summary data set and AD GWAS summary data set. In addition, the genetic variants as instrumental variables in different MR analyses are located in different genes, and linkage disequilibrium has not been proven to have an effect of on potential association analyses. Moreover, we used the three MR methods in this study, which can increase the robustness of the MR results. Several pleiotropic analyses were also conducted, which could reduce the pleiotropic influence on the MR results. We further performed the sensitivity analysis by using the leave-one-out method, which can ensure the stability of the MR results. Finally, the MR studies between the subtypes of stroke and AD were further performed.

Our MR study also has some limitations. Firstly, we only used a small number of genetic variants associated with stroke as the instrumental variable from one GWAS summary data set. Other genetic variants linked with stroke in other studies may not be captured to some extent. Therefore, another stroke GWAS summary data set should be used to identify more genetic variants for an instrumental variable in these MR studies. Secondly, the participants in the AD GWAS summary data set are of European descent. However, the subjects in stroke GWAS summary data set are from trans-ancestral descent, including European descent and other descents. Population stratification may therefore have an effect on the potential association. Thirdly, we tried to figure out several confounder factors of AD, but there may be additional confounder factors that we did not figure out. Fourthly, there may be a little potential pleiotropy when excluding the rs2295786 and rs12445022 in MR-Egger regression during the sensitivity analysis (*P*-value = 0.041 and *P*-value = 0.042, respectively) in the MR analysis between AS and AD.

Although there is some evidence that demonstrated there may be the shared genetics links between AD and ischemic stroke, we found that there is no causal relationship between different kinds of stroke and AD. Our results indicated different types of stroke would not be causally associated with the development of AD.

## Data Availability Statement

Publicly available datasets were analyzed in this study. This data can be found here: AD GWAS summary data set was obtained from the International Genomics of Alzheimer’s Project (IGAP), Stroke GWAS summary data set was obtained from a published article and the GWAS Catalog (study accession: GCST005838).

## Author Contributions

ZH and BS conceived and designed the experiments. TW and QN conducted the experiment. TW and KW wrote the manuscript. All the authors made a contribution to modifying and approving the final version of manuscript.

## Conflict of Interest

The authors declare that the research was conducted in the absence of any commercial or financial relationships that could be construed as a potential conflict of interest.
