# Supplementary material for: Stroke and Alzheimer’s Disease: A Mendelian Randomization Study
Source: Front Genet. 2020 Jul 14;11:581. doi: 10.3389/fgene.2020.00581 (PMC7371994; doi:10.3389/fgene.2020.00581)
Supplement: Supplementary file 1 [file Data_Sheet_1.PDF]

Supplementary-File-1-AS_stroke-and-AD.R

12601

2020-03-27

```
###library packages
library(MendelianRandomization)
```

```
## Warning: package 'MendelianRandomization' was built under R version 3.5.3
```

```
### all 11 SNPs (rs880315, rs12037987, rs16896398, rs7859727, rs2295786, rs35436, rs9526212, rs8103309, rs1052053, rs4959130, rs12445022)
bx <- c(0.0527, 0.071, 0.0477, 0.0494, 0.0526, 0.0462, 0.0587, 0.0501, 0.0624, 0.0779, 0.0574)
bxse <- c(0.0084, 0.0128, 0.0084, 0.0079, 0.0082, 0.0083, 0.0094, 0.0091, 0.0082, 0.0129, 0.0089)

by <- c(-0.0083, -0.0058, -0.0072, 0.024, -0.0263, 0.0112, -0.0187, 0.0249, -0.0209, -0.033, 0.0303)
byse <- c(0.0171, 0.0333, 0.017, 0.0156, 0.0162, 0.0165, 0.0181, 0.0172, 0.016, 0.0236, 0.0167)
### create MRInputObject
MRInputObject <- mr_input(bx = bx,
                          bxse = bxse,
                          by = by,
                          byse = byse)
### output the results for all methods
mr_allmethods(MRInputObject, method = "all")
```

```
##                     Method Estimate Std Error 95% CI        P-value
##              Simple median   -0.151     0.156  -0.457  0.155   0.333
##            Weighted median   -0.156     0.143  -0.436  0.124   0.274
##  Penalized weighted median   -0.202     0.144  -0.485  0.080   0.160
## 
##                        IVW   -0.039     0.122  -0.277  0.200   0.750
##              Penalized IVW   -0.039     0.122  -0.277  0.200   0.750
##                 Robust IVW   -0.060     0.176  -0.406  0.285   0.731
##       Penalized robust IVW   -0.060     0.176  -0.406  0.285   0.731
## 
##                   MR-Egger   -1.312     0.794  -2.868  0.244   0.098
##                (intercept)    0.071     0.044  -0.015  0.157   0.105
##         Penalized MR-Egger   -1.312     0.794  -2.868  0.244   0.098
##                (intercept)    0.071     0.044  -0.015  0.157   0.105
##            Robust MR-Egger   -1.349     0.379  -2.092 -0.606   0.000
##                (intercept)    0.073     0.024   0.026  0.119   0.002
##  Penalized robust MR-Egger   -1.349     0.379  -2.092 -0.606   0.000
##                (intercept)    0.073     0.024   0.026  0.119   0.002
```

```
### output the results for ivw methods, including Heterogeneity test
mr_ivw(MRInputObject)
```

```
##
## Inverse-variance weighted method
## (variants uncorrelated, random-effect model)
##
## Number of Variants : 11
##
## ------------------------------------------------------------------
##  Method Estimate Std Error  95% CI       p-value
##     IVW   -0.039     0.122 -0.277, 0.200   0.750
## ------------------------------------------------------------------
## Residual standard error =  1.260
## Heterogeneity test statistic = 15.8637 on 10 degrees of freedom, (p-value = 0.1036)
```

```
#### remove first SNP
bx1 <- c( 0.071, 0.0477, 0.0494, 0.0526, 0.0462, 0.0587, 0.0501, 0.0624, 0.0779, 0.0574)
bxse1 <- c( 0.0128, 0.0084, 0.0079, 0.0082, 0.0083, 0.0094, 0.0091, 0.0082, 0.0129, 0.0089)

by1 <- c( -0.0058, -0.0072, 0.024, -0.0263, 0.0112, -0.0187, 0.0249, -0.0209, -0.033, 0.0303)
byse1 <- c( 0.0333, 0.017, 0.0156, 0.0162, 0.0165, 0.0181, 0.0172, 0.016, 0.0236, 0.0167)



MRInputObject1 <- mr_input(bx = bx1,
                           bxse = bxse1,
                           by = by1,
                           byse = byse1)

mr_allmethods(MRInputObject1, method = "all")
```

```
##                     Method Estimate Std Error 95% CI         P-value
##              Simple median   -0.116     0.155  -0.420  0.188   0.453
##            Weighted median   -0.192     0.153  -0.492  0.108   0.211
##  Penalized weighted median   -0.311     0.152  -0.610 -0.013   0.041
##
##                        IVW   -0.027     0.134  -0.289  0.235   0.838
##              Penalized IVW   -0.027     0.134  -0.289  0.235   0.838
##                 Robust IVW   -0.040     0.176  -0.385  0.306   0.822
##       Penalized robust IVW   -0.040     0.176  -0.385  0.306   0.822
##
##                   MR-Egger   -1.349     0.831  -2.978  0.280   0.105
##                (intercept)    0.074     0.046  -0.016  0.165   0.108
##         Penalized MR-Egger   -1.349     0.831  -2.978  0.280   0.105
##                (intercept)    0.074     0.046  -0.016  0.165   0.108
##            Robust MR-Egger   -1.378     0.402  -2.166 -0.591   0.001
##                (intercept)    0.076     0.025   0.027  0.125   0.002
##  Penalized robust MR-Egger   -1.378     0.402  -2.166 -0.591   0.001
##                (intercept)    0.076     0.025   0.027  0.125   0.002
```

```r
#### remove second SNP
bx2 <- c(0.0527,  0.0477, 0.0494, 0.0526, 0.0462, 0.0587, 0.0501, 0.0624, 0.0779, 0.0574)
bxse2 <- c(0.0084,  0.0084, 0.0079, 0.0082, 0.0083, 0.0094, 0.0091, 0.0082, 0.0129, 0.0089)

by2 <- c(-0.0083,  -0.0072, 0.024, -0.0263, 0.0112, -0.0187, 0.0249, -0.0209, -0.033, 0.0303)
byse2 <- c(0.0171, 0.017, 0.0156, 0.0162, 0.0165, 0.0181, 0.0172, 0.016, 0.0236, 0.0167)



MRInputObject2 <- mr_input(bx = bx2,
                           bxse = bxse2,
                           by = by2,
                           byse = byse2)

mr_allmethods(MRInputObject2, method = "all")
```

```
##                     Method Estimate Std Error 95% CI        P-value
##              Simple median   -0.154     0.145  -0.438  0.130   0.287
##            Weighted median   -0.167     0.148  -0.456  0.123   0.259
##  Penalized weighted median   -0.247     0.145  -0.531  0.036   0.087
##
##                        IVW   -0.037     0.131  -0.294  0.220   0.778
##              Penalized IVW   -0.037     0.131  -0.294  0.220   0.778
##                 Robust IVW   -0.051     0.170  -0.384  0.281   0.762
##       Penalized robust IVW   -0.051     0.170  -0.384  0.281   0.762
##
##                   MR-Egger   -1.440     0.884  -3.173  0.293   0.103
##                (intercept)    0.078     0.048  -0.017  0.173   0.109
##         Penalized MR-Egger   -1.440     0.884  -3.173  0.293   0.103
##                (intercept)    0.078     0.048  -0.017  0.173   0.109
##            Robust MR-Egger   -1.482     0.391  -2.249 -0.715   0.000
##                (intercept)    0.079     0.023   0.033  0.125   0.001
##  Penalized robust MR-Egger   -1.482     0.391  -2.249 -0.715   0.000
##                (intercept)    0.079     0.023   0.033  0.125   0.001
```

```r
#### remove third SNP
bx3 <- c(0.0527, 0.071,  0.0494, 0.0526, 0.0462, 0.0587, 0.0501, 0.0624, 0.0779, 0.0574)
bxse3 <- c(0.0084, 0.0128,  0.0079, 0.0082, 0.0083, 0.0094, 0.0091, 0.0082, 0.0129, 0.0089)

by3 <- c(-0.0083, -0.0058,  0.024, -0.0263, 0.0112, -0.0187, 0.0249, -0.0209, -0.033, 0.0303)
byse3 <- c(0.0171, 0.0333,  0.0156, 0.0162, 0.0165, 0.0181, 0.0172, 0.016, 0.0236, 0.0167)



MRInputObject3 <- mr_input(bx = bx3,
                           bxse = bxse3,
                           by = by3,
                           byse = byse3)
mr_allmethods(MRInputObject3, method = "all")
```

```
##                     Method Estimate Std Error 95% CI        P-value
##              Simple median   -0.120     0.152  -0.418  0.179   0.433
##            Weighted median   -0.193     0.150  -0.487  0.100   0.197
##  Penalized weighted median   -0.301     0.150  -0.595 -0.007   0.045
##
##                        IVW   -0.030     0.133  -0.290  0.230   0.822
##              Penalized IVW   -0.030     0.133  -0.290  0.230   0.822
##                 Robust IVW   -0.043     0.172  -0.381  0.295   0.805
##       Penalized robust IVW   -0.043     0.172  -0.381  0.295   0.805
##
##                   MR-Egger   -1.527     0.843  -3.180  0.126   0.070
##                (intercept)    0.085     0.047  -0.008  0.177   0.073
##         Penalized MR-Egger   -1.527     0.843  -3.180  0.126   0.070
##                (intercept)    0.085     0.047  -0.008  0.177   0.073
##            Robust MR-Egger   -1.561     0.384  -2.313 -0.808   0.000
##                (intercept)    0.086     0.024   0.039  0.134   0.000
##  Penalized robust MR-Egger   -1.561     0.384  -2.313 -0.808   0.000
##                (intercept)    0.086     0.024   0.039  0.134   0.000
```

```
#### remove forth SNP
bx4 <- c(0.0527, 0.071, 0.0477,  0.0526, 0.0462, 0.0587, 0.0501, 0.0624, 0.0779, 0.0574)
bxse4 <- c(0.0084, 0.0128, 0.0084,  0.0082, 0.0083, 0.0094, 0.0091, 0.0082, 0.0129, 0.0089)

by4 <- c(-0.0083, -0.0058, -0.0072,  -0.0263, 0.0112, -0.0187, 0.0249, -0.0209, -0.033, 0.0303)
byse4 <- c(0.0171, 0.0333, 0.017,  0.0162, 0.0165, 0.0181, 0.0172, 0.016, 0.0236, 0.0167)



MRInputObject4 <- mr_input(bx = bx4,
                           bxse = bxse4,
                           by = by4,
                           byse = byse4)

mr_allmethods(MRInputObject4, method = "all")
```

```
##                     Method Estimate Std Error 95% CI        P-value
##              Simple median   -0.154     0.146  -0.441  0.132   0.292
##            Weighted median   -0.211     0.145  -0.495  0.074   0.147
##  Penalized weighted median   -0.299     0.144  -0.582 -0.017   0.038
##
##                        IVW   -0.093     0.121  -0.330  0.144   0.443
##              Penalized IVW   -0.093     0.121  -0.330  0.144   0.443
##                 Robust IVW   -0.144     0.200  -0.536  0.249   0.473
##       Penalized robust IVW   -0.144     0.200  -0.536  0.249   0.473
##
##                   MR-Egger   -1.097     0.812  -2.688  0.495   0.177
##                (intercept)    0.057     0.045  -0.032  0.146   0.212
##         Penalized MR-Egger   -1.097     0.812  -2.688  0.495   0.177
##                (intercept)    0.057     0.045  -0.032  0.146   0.212
##            Robust MR-Egger   -1.140     0.353  -1.833 -0.448   0.001
##                (intercept)    0.058     0.023   0.012  0.103   0.013
##  Penalized robust MR-Egger   -1.140     0.353  -1.833 -0.448   0.001
##                (intercept)    0.058     0.023   0.012  0.103   0.013
```

```
#### remove fifth SNP
bx5 <- c(0.0527, 0.071, 0.0477, 0.0494,  0.0462, 0.0587, 0.0501, 0.0624, 0.0779, 0.0574)
bxse5 <- c(0.0084, 0.0128, 0.0084, 0.0079,  0.0083, 0.0094, 0.0091, 0.0082, 0.0129, 0.0089)

by5 <- c(-0.0083, -0.0058, -0.0072, 0.024,  0.0112, -0.0187, 0.0249, -0.0209, -0.033, 0.0303)
byse5 <- c(0.0171, 0.0333, 0.017, 0.0156,  0.0165, 0.0181, 0.0172, 0.016, 0.0236, 0.0167)



MRInputObject5 <- mr_input(bx = bx5,
                           bxse = bxse5,
                           by = by5,
                           byse = byse5)

mr_allmethods(MRInputObject5, method = "all")
```

```
##                     Method Estimate Std Error 95% CI        P-value
##              Simple median   -0.116     0.153  -0.416  0.184   0.447
##            Weighted median   -0.152     0.151  -0.447  0.143   0.312
##  Penalized weighted median   -0.155     0.150  -0.450  0.139   0.301
##
##                        IVW    0.011     0.124  -0.232  0.254   0.926
##              Penalized IVW    0.011     0.124  -0.232  0.254   0.926
##                 Robust IVW    0.000     0.167  -0.329  0.328   0.999
##       Penalized robust IVW    0.000     0.167  -0.329  0.328   0.999
##
##                   MR-Egger   -1.432     0.716  -2.836 -0.029   0.046
##                (intercept)    0.081     0.040   0.003  0.159   0.041
##         Penalized MR-Egger   -1.432     0.716  -2.836 -0.029   0.046
##                (intercept)    0.081     0.040   0.003  0.159   0.041
##            Robust MR-Egger   -1.465     0.382  -2.214 -0.715   0.000
##                (intercept)    0.082     0.023   0.037  0.127   0.000
##  Penalized robust MR-Egger   -1.465     0.382  -2.214 -0.715   0.000
##                (intercept)    0.082     0.023   0.037  0.127   0.000
```

```
#### remove sixth SNP
bx6 <- c(0.0527, 0.071, 0.0477, 0.0494, 0.0526,  0.0587, 0.0501, 0.0624, 0.0779, 0.0574)
bxse6 <- c(0.0084, 0.0128, 0.0084, 0.0079, 0.0082,  0.0094, 0.0091, 0.0082, 0.0129, 0.0089)

by6 <- c(-0.0083, -0.0058, -0.0072, 0.024, -0.0263,  -0.0187, 0.0249, -0.0209, -0.033, 0.0303)
byse6 <- c(0.0171, 0.0333, 0.017, 0.0156, 0.0162,  0.0181, 0.0172, 0.016, 0.0236, 0.0167)



MRInputObject6 <- mr_input(bx = bx6,
                           bxse = bxse6,
                           by = by6,
                           byse = byse6)

mr_allmethods(MRInputObject6, method = "all")
```

```
##                     Method Estimate Std Error 95% CI         P-value
##              Simple median   -0.154     0.151  -0.450  0.142   0.307
##            Weighted median   -0.193     0.149  -0.486  0.100   0.196
##  Penalized weighted median   -0.300     0.148  -0.590 -0.011   0.042
##
##                        IVW   -0.061     0.130  -0.316  0.194   0.640
##              Penalized IVW   -0.061     0.130  -0.316  0.194   0.640
##                 Robust IVW   -0.120     0.251  -0.612  0.371   0.631
##       Penalized robust IVW   -0.120     0.251  -0.612  0.371   0.631
##
##                   MR-Egger   -1.299     0.907  -3.076  0.478   0.152
##                (intercept)    0.070     0.051  -0.030  0.170   0.168
##         Penalized MR-Egger   -1.299     0.907  -3.076  0.478   0.152
##                (intercept)    0.070     0.051  -0.030  0.170   0.168
##            Robust MR-Egger   -1.335     0.518  -2.350 -0.319   0.010
##                (intercept)    0.071     0.035   0.003  0.140   0.042
##  Penalized robust MR-Egger   -1.335     0.518  -2.350 -0.319   0.010
##                (intercept)    0.071     0.035   0.003  0.140   0.042
```

```
#### remove seventh SNP
bx7 <- c(0.0527, 0.071, 0.0477, 0.0494, 0.0526, 0.0462,  0.0501, 0.0624, 0.0779, 0.0574)
bxse7 <- c(0.0084, 0.0128, 0.0084, 0.0079, 0.0082, 0.0083,  0.0091, 0.0082, 0.0129, 0.0089)

by7 <- c(-0.0083, -0.0058, -0.0072, 0.024, -0.0263, 0.0112,  0.0249, -0.0209, -0.033, 0.0303)
byse7 <- c(0.0171, 0.0333, 0.017, 0.0156, 0.0162, 0.0165,  0.0172, 0.016, 0.0236, 0.0167)



MRInputObject7 <- mr_input(bx = bx7,
                           bxse = bxse7,
                           by = by7,
                           byse = byse7)

mr_allmethods(MRInputObject7, method = "all")
```

```
##                     Method Estimate Std Error 95% CI         P-value
##              Simple median   -0.116     0.158  -0.427  0.194   0.463
##            Weighted median   -0.152     0.155  -0.455  0.151   0.325
##  Penalized weighted median   -0.155     0.153  -0.455  0.144   0.309
##
##                        IVW   -0.008     0.131  -0.265  0.248   0.949
##              Penalized IVW   -0.008     0.131  -0.265  0.248   0.949
##                 Robust IVW   -0.016     0.159  -0.327  0.296   0.921
##       Penalized robust IVW   -0.016     0.159  -0.327  0.296   0.921
##
##                   MR-Egger   -1.232     0.833  -2.864  0.401   0.139
##                (intercept)    0.068     0.046  -0.022  0.158   0.138
##         Penalized MR-Egger   -1.232     0.833  -2.864  0.401   0.139
##                (intercept)    0.068     0.046  -0.022  0.158   0.138
##            Robust MR-Egger   -1.262     0.396  -2.038 -0.486   0.001
##                (intercept)    0.069     0.024   0.023  0.116   0.003
##  Penalized robust MR-Egger   -1.262     0.396  -2.038 -0.486   0.001
##                (intercept)    0.069     0.024   0.023  0.116   0.003
```

```r
#### remove eighth SNP
bx8 <- c(0.0527, 0.071, 0.0477, 0.0494, 0.0526, 0.0462, 0.0587,  0.0624, 0.0779, 0.0574)
bxse8 <- c(0.0084, 0.0128, 0.0084, 0.0079, 0.0082, 0.0083, 0.0094,  0.0082, 0.0129, 0.0089)

by8 <- c(-0.0083, -0.0058, -0.0072, 0.024, -0.0263, 0.0112, -0.0187,  -0.0209, -0.033, 0.0303)
byse8 <- c(0.0171, 0.0333, 0.017, 0.0156, 0.0162, 0.0165, 0.0181,  0.016, 0.0236, 0.0167)



MRInputObject8 <- mr_input(bx = bx8,
                           bxse = bxse8,
                           by = by8,
                           byse = byse8)

mr_allmethods(MRInputObject8, method = "all")
```

```
##                     Method Estimate Std Error 95% CI        P-value
##              Simple median   -0.154     0.147  -0.442  0.134   0.294
##            Weighted median   -0.198     0.146  -0.485  0.088   0.175
##  Penalized weighted median   -0.301     0.145  -0.586 -0.017   0.038
##
##                        IVW   -0.085     0.122  -0.324  0.154   0.487
##              Penalized IVW   -0.085     0.122  -0.324  0.154   0.487
##                 Robust IVW   -0.138     0.211  -0.553  0.276   0.513
##       Penalized robust IVW   -0.138     0.211  -0.553  0.276   0.513
##
##                   MR-Egger   -1.150     0.805  -2.727  0.427   0.153
##                (intercept)    0.060     0.045  -0.028  0.148   0.181
##         Penalized MR-Egger   -1.150     0.805  -2.727  0.427   0.153
##                (intercept)    0.060     0.045  -0.028  0.148   0.181
##            Robust MR-Egger   -1.192     0.372  -1.921 -0.463   0.001
##                (intercept)    0.061     0.025   0.013  0.109   0.013
##  Penalized robust MR-Egger   -1.192     0.372  -1.921 -0.463   0.001
##                (intercept)    0.061     0.025   0.013  0.109   0.013
```

```r
#### remove nineth SNP
bx9 <- c(0.0527, 0.071, 0.0477, 0.0494, 0.0526, 0.0462, 0.0587, 0.0501,  0.0779, 0.0574)
bxse9 <- c(0.0084, 0.0128, 0.0084, 0.0079, 0.0082, 0.0083, 0.0094, 0.0091,  0.0129, 0.0089)

by9 <- c(-0.0083, -0.0058, -0.0072, 0.024, -0.0263, 0.0112, -0.0187, 0.0249,  -0.033, 0.0303)
byse9 <- c(0.0171, 0.0333, 0.017, 0.0156, 0.0162, 0.0165, 0.0181, 0.0172,  0.0236, 0.0167)



MRInputObject9 <- mr_input(bx = bx9,
                           bxse = bxse9,
                           by = by9,
                           byse = byse9)

mr_allmethods(MRInputObject9, method = "all")
```

```
##                    Method Estimate Std Error 95% CI        P-value
##             Simple median   -0.116     0.158  -0.426  0.193   0.461
##           Weighted median   -0.144     0.156  -0.450  0.163   0.357
##  Penalized weighted median  -0.153     0.158  -0.464  0.157   0.333
##
##                       IVW    0.010     0.131  -0.247  0.268   0.939
##             Penalized IVW    0.010     0.131  -0.247  0.268   0.939
##                Robust IVW    0.004     0.157  -0.303  0.312   0.979
##      Penalized robust IVW    0.004     0.157  -0.303  0.312   0.979
##
##                  MR-Egger   -1.133     0.879  -2.855  0.589   0.197
##               (intercept)    0.063     0.048  -0.031  0.156   0.189
##        Penalized MR-Egger   -1.133     0.879  -2.855  0.589   0.197
##               (intercept)    0.063     0.048  -0.031  0.156   0.189
##           Robust MR-Egger   -1.166     0.407  -1.965 -0.368   0.004
##               (intercept)    0.064     0.023   0.018  0.110   0.006
##  Penalized robust MR-Egger  -1.166     0.407  -1.965 -0.368   0.004
##               (intercept)    0.064     0.023   0.018  0.110   0.006
```

```
#### remove tenth SNP
bx10 <- c(0.0527, 0.071, 0.0477, 0.0494, 0.0526, 0.0462, 0.0587, 0.0501, 0.0624,  0.0574)
bxse10 <- c(0.0084, 0.0128, 0.0084, 0.0079, 0.0082, 0.0083, 0.0094, 0.0091, 0.0082,  0.0089)

by10 <- c(-0.0083, -0.0058, -0.0072, 0.024, -0.0263, 0.0112, -0.0187, 0.0249, -0.0209,  0.0303)
byse10 <- c(0.0171, 0.0333, 0.017, 0.0156, 0.0162, 0.0165, 0.0181, 0.0172, 0.016,  0.0167)



MRInputObject10 <- mr_input(bx = bx10,
                            bxse = bxse10,
                            by = by10,
                            byse = byse10)

mr_allmethods(MRInputObject10, method = "all")
```

```
##                    Method Estimate Std Error 95% CI       P-value
##             Simple median   -0.116     0.155  -0.420 0.187   0.453
##           Weighted median   -0.152     0.153  -0.452 0.147   0.319
##  Penalized weighted median  -0.155     0.152  -0.453 0.143   0.307
##
##                       IVW    0.005     0.127  -0.245 0.254   0.971
##             Penalized IVW    0.005     0.127  -0.245 0.254   0.971
##                Robust IVW   -0.003     0.158  -0.314 0.307   0.983
##      Penalized robust IVW   -0.003     0.158  -0.314 0.307   0.983
##
##                  MR-Egger   -1.240     1.156  -3.505 1.025   0.283
##               (intercept)    0.067     0.062  -0.054 0.189   0.279
##        Penalized MR-Egger   -1.240     1.156  -3.505 1.025   0.283
##               (intercept)    0.067     0.062  -0.054 0.189   0.279
##           Robust MR-Egger   -1.301     0.743  -2.758 0.156   0.080
##               (intercept)    0.070     0.039  -0.007 0.148   0.075
##  Penalized robust MR-Egger  -1.301     0.743  -2.758 0.156   0.080
##               (intercept)    0.070     0.039  -0.007 0.148   0.075
```

```r
#### remove eleventh SNP
bx11 <- c(0.0527, 0.071, 0.0477, 0.0494, 0.0526, 0.0462, 0.0587, 0.0501, 0.0624, 0.0779)
bxse11 <- c(0.0084, 0.0128, 0.0084, 0.0079, 0.0082, 0.0083, 0.0094, 0.0091, 0.0082, 0.0129)

by11 <- c(-0.0083, -0.0058, -0.0072, 0.024, -0.0263, 0.0112, -0.0187, 0.0249, -0.0209, -0.033)
byse11 <- c(0.0171, 0.0333, 0.017, 0.0156, 0.0162, 0.0165, 0.0181, 0.0172, 0.016, 0.0236)



MRInputObject11 <- mr_input(bx = bx11,
                            bxse = bxse11,
                            by = by11,
                            byse = byse11)

mr_allmethods(MRInputObject11, method = "all")
```

```
##                    Method Estimate Std Error 95% CI        P-value
##             Simple median   -0.154     0.147  -0.442  0.133   0.293
##           Weighted median   -0.225     0.144  -0.508  0.058   0.119
##  Penalized weighted median   -0.286     0.144  -0.569 -0.003   0.048
##
##                       IVW   -0.109     0.116  -0.337  0.119   0.348
##             Penalized IVW   -0.109     0.116  -0.337  0.119   0.348
##                Robust IVW   -0.147     0.175  -0.490  0.197   0.403
##      Penalized robust IVW   -0.147     0.175  -0.490  0.197   0.403
##
##                  MR-Egger   -1.485     0.684  -2.826 -0.144   0.030
##               (intercept)    0.077     0.038   0.003  0.150   0.042
##        Penalized MR-Egger   -1.485     0.684  -2.826 -0.144   0.030
##               (intercept)    0.077     0.038   0.003  0.150   0.042
##           Robust MR-Egger   -1.458     0.419  -2.279 -0.637   0.001
##               (intercept)    0.075     0.027   0.022  0.128   0.006
##  Penalized robust MR-Egger   -1.458     0.419  -2.279 -0.637   0.001
##               (intercept)    0.075     0.027   0.022  0.128   0.006
```

```r
##install R package TwoSampleMR
# library(devtools)
# devtools::install_github("MRCIEU/TwoSampleMR@0.4.26")

##library R package
library(TwoSampleMR)
```

```
## Welcome to TwoSampleMR.
## [>] Full documentation: https://mrcieu.github.io/TwoSampleMR
## [>] Check news(package='TwoSampleMR') for bug fixes and updates
## [>] By generating access tokens to retrieve data from the MR-Base
##     database you consent to having your email address logged on
##     our servers. For info on how this is used see logging_info()
## [>] NOTE: We will be rolling out extensive changes to the database
##     in the next few weeks. To ensure backwards compatibility please
##     keep the R package updated.
```

```
##
## Warning:
## You are running an old version of the TwoSampleMR package.
## This version:   0.4.26
## Latest version: 0.5.2
## Please consider updating using devtools::install_github('MRCIEU/TwoSampleMR')
```

```
##
## Attaching package: 'TwoSampleMR'
```

```
## The following objects are masked from 'package:MendelianRandomization':
##
##     mr_ivw, mr_median
```

```
### read exposure data (11 SNPs associated with any stroke)
any_stroke_dat <- read_exposure_data("C:/Users/12601/Desktop/MR_modifition/TwoSampleMR_exposure
AS and AD.txt")

###print exposure data
any_stroke_dat
```

```
##           SNP beta.exposure se.exposure effect_allele.exposure
## 1    rs880315        0.0527      0.0084                      C
## 2  rs12037987        0.0710      0.0128                      C
## 3  rs16896398        0.0477      0.0084                      T
## 4   rs7859727        0.0494      0.0079                      T
## 5   rs2295786        0.0526      0.0082                      A
## 6     rs35436        0.0462      0.0083                      C
## 7   rs9526212        0.0587      0.0094                      G
## 8   rs8103309        0.0501      0.0091                      T
## 9   rs1052053        0.0624      0.0082                      G
## 10  rs4959130        0.0779      0.0129                      A
## 11 rs12445022        0.0574      0.0089                      A
##    other_allele.exposure eaf.exposure pval.exposure  gene.exposure
## 1                      T         0.40      3.62e-10          CASZ1
## 2                      T         0.16      2.73e-08          WNT2B
## 3                      A         0.34      1.30e-08 SLC22A7-ZNF318
## 4                      C         0.53      4.22e-10        Chr9p21
## 5                      T         0.60      1.80e-10       SH3PXD2A
## 6                      T         0.62      2.87e-08           TBX3
## 7                      A         0.76      5.03e-10          LRCH1
## 8                      C         0.65      3.40e-08   SMARCA4-LDLR
## 9                      A         0.40      2.70e-14    PMF1-SEMA4A
## 10                     G         0.14      1.42e-09          FOXF2
## 11                     G         0.31      1.05e-10        ZCCHC14
##    samplesize.exposure   exposure mr_keep.exposure pval_origin.exposure
## 1               521612 Any_Stroke             TRUE             reported
## 2               521612 Any_Stroke             TRUE             reported
## 3               521612 Any_Stroke             TRUE             reported
## 4               521612 Any_Stroke             TRUE             reported
## 5               521612 Any_Stroke             TRUE             reported
## 6               521612 Any_Stroke             TRUE             reported
## 7               521612 Any_Stroke             TRUE             reported
## 8               521612 Any_Stroke             TRUE             reported
## 9               521612 Any_Stroke             TRUE             reported
## 10              521612 Any_Stroke             TRUE             reported
## 11              521612 Any_Stroke             TRUE             reported
##    id.exposure data_source.exposure
## 1       LLOMZy             textfile
## 2       LLOMZy             textfile
## 3       LLOMZy             textfile
## 4       LLOMZy             textfile
## 5       LLOMZy             textfile
## 6       LLOMZy             textfile
## 7       LLOMZy             textfile
## 8       LLOMZy             textfile
## 9       LLOMZy             textfile
## 10      LLOMZy             textfile
## 11      LLOMZy             textfile
```

```
### read outcome data (11 SNPs from AD GWAS)
AD_outcome_dat <- read_outcome_data(snps = any_stroke_dat$SNP,
                                    filename = "C:/Users/12601/Desktop/MR_modifition/TwoSampleM
R_outcome AS and AD.csv",
                                    sep = ",",snp_col = "SNP",beta_col = "beta",se_col = "se",
                                    effect_allele_col = "effect_allele",other_allele_col = "oth
er_allele",
                                    gene_col = "gene",samplesize_col = "samplesize")
```

```
## Warning in format_data(as.data.frame(outcome_dat), type = "outcome", snps = snps, : The foll
owing columns are not present but are helpful for harmonisation
## eaf
```

```
###print outcome data
AD_outcome_dat
```

```
##           SNP beta.outcome se.outcome effect_allele.outcome
## 1    rs880315      -0.0083     0.0171                     C
## 2  rs12037987      -0.0058     0.0333                     C
## 3  rs16896398      -0.0072     0.0170                     T
## 4   rs7859727       0.0240     0.0156                     T
## 5   rs2295786      -0.0263     0.0162                     A
## 6     rs35436       0.0112     0.0165                     C
## 7   rs9526212      -0.0187     0.0181                     G
## 8   rs8103309       0.0249     0.0172                     T
## 9   rs1052053      -0.0209     0.0160                     G
## 10  rs4959130      -0.0330     0.0236                     A
## 11 rs12445022       0.0303     0.0167                     A
##    other_allele.outcome pval.outcome   gene.outcome samplesize.outcome outcome
## 1                     T      0.63010          CASZ1              54162      AD
## 2                     T      0.86060          WNT2B              54162      AD
## 3                     A      0.67190 SLC22A7-ZNF318              54162      AD
## 4                     C      0.12440        Chr9p21              54162      AD
## 5                     T      0.10430       SH3PXD2A              54162      AD
## 6                     T      0.49490           TBX3              54162      AD
## 7                     A      0.30150          LRCH1              54162      AD
## 8                     C      0.14870   SMARCA4-LDLR              54162      AD
## 9                     A      0.18980    PMF1-SEMA4A              54162      AD
## 10                    G      0.16250          FOXF2              54162      AD
## 11                    G      0.07066        ZCCHC14              54162      AD
##    mr_keep.outcome pval_origin.outcome id.outcome eaf.outcome
## 1             TRUE            reported     bfN2YJ          NA
## 2             TRUE            reported     bfN2YJ          NA
## 3             TRUE            reported     bfN2YJ          NA
## 4             TRUE            reported     bfN2YJ          NA
## 5             TRUE            reported     bfN2YJ          NA
## 6             TRUE            reported     bfN2YJ          NA
## 7             TRUE            reported     bfN2YJ          NA
## 8             TRUE            reported     bfN2YJ          NA
## 9             TRUE            reported     bfN2YJ          NA
## 10            TRUE            reported     bfN2YJ          NA
## 11            TRUE            reported     bfN2YJ          NA
##    data_source.outcome
## 1             textfile
## 2             textfile
## 3             textfile
## 4             textfile
## 5             textfile
## 6             textfile
## 7             textfile
## 8             textfile
## 9             textfile
## 10            textfile
## 11            textfile
```

```
### harmonise exposure data and outcome data
dat <- harmonise_data(any_stroke_dat, AD_outcome_dat)
```

```
## Harmonising Any_Stroke (LLOMZy) and AD (bfN2YJ)
```

```
## Removing the following SNPs for being palindromic with intermediate allele frequencies:
## rs16896398, rs2295786
```

```
### set up unit for the exposure
dat$units.exposure <- "OR"

### set up unit for the outcome
dat$units.outcome <- "OR"

### run Steiger filtering for each SNP
dat2 <- steiger_filtering(dat)
```

```
## Estimating correlation for quantitative trait.
```

```
## This method is an approximation, and may be numerically unstable.
```

```
## Ideally you should estimate r directly from independent replication samples.
```

```
## Use get_r_from_lor for binary traits.
```

```
## Estimating correlation for quantitative trait.
```

```
## This method is an approximation, and may be numerically unstable.
```

```
## Ideally you should estimate r directly from independent replication samples.
```

```
## Use get_r_from_lor for binary traits.
```

```
### MR analysis excluding instruments with the wrong direction of effects
mr_results <- mr(subset(dat2,steiger_dir))
```

```
## Analysing 'LLOMZy' on 'bfN2YJ'
```

```
### print mr_results
mr_results
```

```
##   id.exposure id.outcome outcome   exposure                    method nsnp
## 1      LLOMZy     bfN2YJ      AD Any_Stroke                  MR Egger    9
## 2      LLOMZy     bfN2YJ      AD Any_Stroke           Weighted median    9
## 3      LLOMZy     bfN2YJ      AD Any_Stroke Inverse variance weighted    9
## 4      LLOMZy     bfN2YJ      AD Any_Stroke               Simple mode    9
## 5      LLOMZy     bfN2YJ      AD Any_Stroke             Weighted mode    9
##             b        se       pval
## 1 -1.72498155 0.7217316 0.04816458
## 2 -0.12902321 0.1518867 0.39561970
## 3  0.02586409 0.1360326 0.84920611
## 4 -0.25334294 0.3463685 0.48538906
## 5 -0.29407230 0.3001269 0.35586959
```
