# Supplementary material for: Stroke and Alzheimer’s Disease: A Mendelian Randomization Study
Source: Front Genet. 2020 Jul 14;11:581. doi: 10.3389/fgene.2020.00581 (PMC7371994; doi:10.3389/fgene.2020.00581)
Supplement: Supplementary file 2 [file Data_Sheet_2.PDF]

Supplementary-File-2-AIS_stroke-and-AD.R

12601

2020-03-27

```r
###library packages
library(MendelianRandomization)
```

```
## Warning: package 'MendelianRandomization' was built under R version 3.5.3
```

```r
### all 9 SNPs(rs6825454,rs11957829,rs7304841,rs4932370,rs11867415,rs2229383,rs635634,rs2005108,rs3184504)
bx <- c(0.0583,0.0677,0.0488,0.0488,0.0862,0.0488,0.0770,0.0770,0.0770)
bxse <- c(0.0096,0.0119,0.0097,0.0097,0.0163,0.0097,0.0142,0.0142,0.0094)

by <- c(0.0047,0.0093,-0.0142,-0.0071,-0.0392,0.0158,-0.0055,-0.0003,-0.0252)
byse <- c(0.0184,0.022,0.0176,0.0171,0.0347,0.0169,0.0195,0.0231,0.0159)
### create MRInputObject
MRInputObject <- mr_input(bx = bx,
                          bxse = bxse,
                          by = by,
                          byse = byse)
### output the results for all methods
mr_allmethods(MRInputObject, method = "all")
```

```
##                     Method Estimate Std Error 95% CI        P-value
##              Simple median   -0.071     0.141  -0.347 0.204   0.611
##            Weighted median   -0.092     0.132  -0.350 0.167   0.487
##  Penalized weighted median   -0.092     0.132  -0.350 0.167   0.487
##
##                        IVW   -0.103     0.100  -0.298 0.093   0.305
##              Penalized IVW   -0.103     0.100  -0.298 0.093   0.305
##                 Robust IVW   -0.101     0.087  -0.271 0.069   0.242
##       Penalized robust IVW   -0.101     0.087  -0.271 0.069   0.242
##
##                   MR-Egger   -0.512     0.485  -1.462 0.437   0.290
##                (intercept)    0.027     0.031  -0.034 0.088   0.387
##         Penalized MR-Egger   -0.512     0.485  -1.462 0.437   0.290
##                (intercept)    0.027     0.031  -0.034 0.088   0.387
##            Robust MR-Egger   -0.509     0.363  -1.221 0.203   0.161
##                (intercept)    0.027     0.024  -0.020 0.074   0.266
##  Penalized robust MR-Egger   -0.509     0.363  -1.221 0.203   0.161
##                (intercept)    0.027     0.024  -0.020 0.074   0.266
```

```r
### output the results for ivw methods, including Heterogeneity test
mr_ivw(MRInputObject)
```

```
##
## Inverse-variance weighted method
## (variants uncorrelated, random-effect model)
##
## Number of Variants : 9
##
## ------------------------------------------------------------------
##  Method Estimate Std Error  95% CI       p-value
##     IVW   -0.103     0.100 -0.298, 0.093   0.305
## ------------------------------------------------------------------
## Residual standard error =  0.771
## Residual standard error is set to 1 in calculation of confidence interval when its estimate is less than 1.
## Heterogeneity test statistic = 4.7585 on 8 degrees of freedom, (p-value = 0.7831)
```

```
#remove first SNP
bx1 <- c(0.0677,0.0488,0.0488,0.0862,0.0488,0.0770,0.0770,0.0770)
bxse1 <- c(0.0119,0.0097,0.0097,0.0163,0.0097,0.0142,0.0142,0.0094)

by1 <- c(0.0093,-0.0142,-0.0071,-0.0392,0.0158,-0.0055,-0.0003,-0.0252)
byse1 <- c(0.022,0.0176,0.0171,0.0347,0.0169,0.0195,0.0231,0.0159)



MRInputObject1 <- mr_input(bx = bx1,
                          bxse = bxse1,
                          by = by1,
                          byse = byse1)

mr_allmethods(MRInputObject1, method = "all")
```

```
##                     Method Estimate Std Error 95% CI        P-value
##              Simple median   -0.108     0.139  -0.380 0.163   0.434
##            Weighted median   -0.123     0.138  -0.393 0.147   0.373
##  Penalized weighted median   -0.123     0.138  -0.393 0.147   0.373
##
##                        IVW   -0.123     0.105  -0.330 0.084   0.244
##              Penalized IVW   -0.123     0.105  -0.330 0.084   0.244
##                 Robust IVW   -0.125     0.086  -0.293 0.043   0.146
##       Penalized robust IVW   -0.125     0.086  -0.293 0.043   0.146
##
##                  MR-Egger   -0.486     0.488  -1.443 0.471   0.320
##                (intercept)    0.024     0.032  -0.038 0.086   0.446
##         Penalized MR-Egger   -0.486     0.488  -1.443 0.471   0.320
##                (intercept)    0.024     0.032  -0.038 0.086   0.446
##            Robust MR-Egger   -0.479     0.362  -1.189 0.231   0.186
##                (intercept)    0.024     0.024  -0.024 0.071   0.335
##  Penalized robust MR-Egger   -0.479     0.362  -1.189 0.231   0.186
##                (intercept)    0.024     0.024  -0.024 0.071   0.335
```

```r
#remove second SNP
bx2 <- c(0.0583,0.0488,0.0488,0.0862,0.0488,0.0770,0.0770,0.0770)
bxse2 <- c(0.0096,0.0097,0.0097,0.0163,0.0097,0.0142,0.0142,0.0094)

by2 <- c(0.0047,-0.0142,-0.0071,-0.0392,0.0158,-0.0055,-0.0003,-0.0252)
byse2 <- c(0.0184,0.0176,0.0171,0.0347,0.0169,0.0195,0.0231,0.0159)



MRInputObject2 <- mr_input(bx = bx2,
                          bxse = bxse2,
                          by = by2,
                          byse = byse2)

mr_allmethods(MRInputObject2, method = "all")
```

```
##                     Method Estimate Std Error 95% CI        P-value
##              Simple median   -0.108     0.138  -0.378 0.161   0.431
##            Weighted median   -0.121     0.137  -0.390 0.147   0.376
##  Penalized weighted median   -0.121     0.137  -0.390 0.147   0.376
##
##                        IVW   -0.128     0.105  -0.334 0.078   0.225
##              Penalized IVW   -0.128     0.105  -0.334 0.078   0.225
##                 Robust IVW   -0.130     0.086  -0.299 0.040   0.134
##        Penalized robust IVW   -0.130     0.086  -0.299 0.040   0.134
##
##                   MR-Egger   -0.560     0.488  -1.516 0.397   0.251
##                (intercept)    0.028     0.031  -0.033 0.089   0.364
##         Penalized MR-Egger   -0.560     0.488  -1.516 0.397   0.251
##                (intercept)    0.028     0.031  -0.033 0.089   0.364
##            Robust MR-Egger   -0.557     0.349  -1.242 0.128   0.111
##                (intercept)    0.028     0.023  -0.018 0.074   0.230
##  Penalized robust MR-Egger   -0.557     0.349  -1.242 0.128   0.111
##                (intercept)    0.028     0.023  -0.018 0.074   0.230
```

```r
### remove third SNP
bx3 <- c(0.0583,0.0677,0.0488,0.0862,0.0488,0.0770,0.0770,0.0770)
bxse3 <- c(0.0096,0.0119,0.0097,0.0163,0.0097,0.0142,0.0142,0.0094)

by3 <- c(0.0047,0.0093,-0.0071,-0.0392,0.0158,-0.0055,-0.0003,-0.0252)
byse3 <- c(0.0184,0.022,0.0171,0.0347,0.0169,0.0195,0.0231,0.0159)



MRInputObject3 <- mr_input(bx = bx3,
                          bxse = bxse3,
                          by = by3,
                          byse = byse3)
mr_allmethods(MRInputObject3, method = "all")
```

```
##                     Method Estimate Std Error 95% CI        P-value
##              Simple median   -0.038     0.137  -0.307 0.232   0.784
##            Weighted median   -0.068     0.137  -0.336 0.199   0.616
##  Penalized weighted median   -0.068     0.137  -0.336 0.199   0.616
##
##                        IVW   -0.087     0.104  -0.291 0.117   0.404
##              Penalized IVW   -0.087     0.104  -0.291 0.117   0.404
##                 Robust IVW   -0.082     0.102  -0.283 0.119   0.423
##       Penalized robust IVW   -0.082     0.102  -0.283 0.119   0.423
##
##                   MR-Egger   -0.743     0.531  -1.783 0.297   0.162
##                (intercept)    0.044     0.035  -0.024 0.113   0.207
##         Penalized MR-Egger   -0.743     0.531  -1.783 0.297   0.162
##                (intercept)    0.044     0.035  -0.024 0.113   0.207
##            Robust MR-Egger   -0.752     0.423  -1.582 0.078   0.076
##                (intercept)    0.045     0.028  -0.011 0.100   0.113
##  Penalized robust MR-Egger   -0.752     0.423  -1.582 0.078   0.076
##                (intercept)    0.045     0.028  -0.011 0.100   0.113
```

```
### remove forth SNP
bx4 <- c(0.0583,0.0677,0.0488,0.0862,0.0488,0.0770,0.0770,0.0770)
bxse4 <- c(0.0096,0.0119,0.0097,0.0163,0.0097,0.0142,0.0142,0.0094)

by4 <- c(0.0047,0.0093,-0.0142,-0.0392,0.0158,-0.0055,-0.0003,-0.0252)
byse4 <- c(0.0184,0.022,0.0176,0.0347,0.0169,0.0195,0.0231,0.0159)



MRInputObject4 <- mr_input(bx = bx4,
                          bxse = bxse4,
                          by = by4,
                          byse = byse4)

mr_allmethods(MRInputObject4, method = "all")
```

```
##                     Method Estimate Std Error 95% CI        P-value
##              Simple median   -0.038     0.138  -0.308 0.233   0.785
##            Weighted median   -0.067     0.138  -0.337 0.202   0.625
##  Penalized weighted median   -0.067     0.138  -0.337 0.202   0.625
##
##                        IVW   -0.099     0.104  -0.303 0.106   0.344
##              Penalized IVW   -0.099     0.104  -0.303 0.106   0.344
##                 Robust IVW   -0.097     0.090  -0.273 0.079   0.281
##       Penalized robust IVW   -0.097     0.090  -0.273 0.079   0.281
##
##                   MR-Egger   -0.651     0.534  -1.698 0.396   0.223
##                (intercept)    0.037     0.035  -0.032 0.106   0.292
##         Penalized MR-Egger   -0.651     0.534  -1.698 0.396   0.223
##                (intercept)    0.037     0.035  -0.032 0.106   0.292
##            Robust MR-Egger   -0.681     0.527  -1.715 0.352   0.196
##                (intercept)    0.040     0.037  -0.033 0.113   0.286
##  Penalized robust MR-Egger   -0.681     0.527  -1.715 0.352   0.196
##                (intercept)    0.040     0.037  -0.033 0.113   0.286
```

```r
### remove fifth SNP
bx5 <- c(0.0583,0.0677,0.0488,0.0488,0.0488,0.0770,0.0770,0.0770)
bxse5 <- c(0.0096,0.0119,0.0097,0.0097,0.0097,0.0142,0.0142,0.0094)

by5 <- c(0.0047,0.0093,-0.0142,-0.0071,0.0158,-0.0055,-0.0003,-0.0252)
byse5 <- c(0.0184,0.022,0.0176,0.0171,0.0169,0.0195,0.0231,0.0159)



MRInputObject5 <- mr_input(bx = bx5,
                           bxse = bxse5,
                           by = by5,
                           byse = byse5)

mr_allmethods(MRInputObject5, method = "all")
```

```
##                     Method Estimate Std Error 95% CI        P-value
##              Simple median   -0.038     0.137  -0.305 0.230   0.783
##            Weighted median   -0.072     0.136  -0.338 0.193   0.593
##  Penalized weighted median   -0.072     0.136  -0.338 0.193   0.593
##
##                        IVW   -0.079     0.103  -0.282 0.123   0.442
##              Penalized IVW   -0.079     0.103  -0.282 0.123   0.442
##                 Robust IVW   -0.074     0.107  -0.284 0.136   0.490
##       Penalized robust IVW   -0.074     0.107  -0.284 0.136   0.490
##
##                   MR-Egger   -0.395     0.514  -1.403 0.613   0.443
##                (intercept)    0.020     0.032  -0.043 0.084   0.531
##         Penalized MR-Egger   -0.395     0.514  -1.403 0.613   0.443
##                (intercept)    0.020     0.032  -0.043 0.084   0.531
##            Robust MR-Egger   -0.375     0.466  -1.289 0.539   0.421
##                (intercept)    0.019     0.029  -0.039 0.077   0.514
##  Penalized robust MR-Egger   -0.375     0.466  -1.289 0.539   0.421
##                (intercept)    0.019     0.029  -0.039 0.077   0.514
```

```r
### remove sixth SNP
bx6 <- c(0.0583,0.0677,0.0488,0.0488,0.0862,0.0770,0.0770,0.0770)
bxse6 <- c(0.0096,0.0119,0.0097,0.0097,0.0163,0.0142,0.0142,0.0094)

by6 <- c(0.0047,0.0093,-0.0142,-0.0071,-0.0392,-0.0055,-0.0003,-0.0252)
byse6 <- c(0.0184,0.022,0.0176,0.0171,0.0347,0.0195,0.0231,0.0159)



MRInputObject6 <- mr_input(bx = bx6,
                           bxse = bxse6,
                           by = by6,
                           byse = byse6)

mr_allmethods(MRInputObject6, method = "all")
```

```
##                     Method Estimate Std Error 95% CI        P-value
##              Simple median   -0.108     0.135  -0.373 0.156   0.421
##            Weighted median   -0.118     0.133  -0.379 0.144   0.377
##  Penalized weighted median   -0.118     0.133  -0.379 0.144   0.377
##
##                        IVW   -0.141     0.104  -0.346 0.063   0.176
##              Penalized IVW   -0.141     0.104  -0.346 0.063   0.176
##                 Robust IVW   -0.139     0.093  -0.320 0.042   0.133
##       Penalized robust IVW   -0.139     0.093  -0.320 0.042   0.133
##
##                   MR-Egger   -0.286     0.536  -1.336 0.764   0.593
##                (intercept)    0.010     0.035  -0.060 0.079   0.783
##         Penalized MR-Egger   -0.286     0.536  -1.336 0.764   0.593
##                (intercept)    0.010     0.035  -0.060 0.079   0.783
##            Robust MR-Egger   -0.274     0.351  -0.962 0.414   0.435
##                (intercept)    0.009     0.021  -0.033 0.051   0.672
##  Penalized robust MR-Egger   -0.274     0.351  -0.962 0.414   0.435
##                (intercept)    0.009     0.021  -0.033 0.051   0.672
```

```
### remove seventh SNP
bx7 <- c(0.0583,0.0677,0.0488,0.0488,0.0862,0.0488,0.0770,0.0770)
bxse7 <- c(0.0096,0.0119,0.0097,0.0097,0.0163,0.0097,0.0142,0.0094)

by7 <- c(0.0047,0.0093,-0.0142,-0.0071,-0.0392,0.0158,-0.0003,-0.0252)
byse7 <- c(0.0184,0.022,0.0176,0.0171,0.0347,0.0169,0.0231,0.0159)



MRInputObject7 <- mr_input(bx = bx7,
                          bxse = bxse7,
                          by = by7,
                          byse = byse7)

mr_allmethods(MRInputObject7, method = "all")
```

```
##                     Method Estimate Std Error 95% CI        P-value
##              Simple median   -0.075     0.144  -0.358 0.208   0.605
##            Weighted median   -0.133     0.144  -0.416 0.149   0.355
##  Penalized weighted median   -0.133     0.144  -0.416 0.149   0.355
##
##                        IVW   -0.108     0.109  -0.322 0.105   0.320
##              Penalized IVW   -0.108     0.109  -0.322 0.105   0.320
##                 Robust IVW   -0.108     0.097  -0.298 0.081   0.263
##       Penalized robust IVW   -0.108     0.097  -0.298 0.081   0.263
##
##                   MR-Egger   -0.596     0.524  -1.623 0.431   0.255
##                (intercept)    0.031     0.033  -0.033 0.095   0.341
##         Penalized MR-Egger   -0.596     0.524  -1.623 0.431   0.255
##                (intercept)    0.031     0.033  -0.033 0.095   0.341
##            Robust MR-Egger   -0.600     0.386  -1.356 0.156   0.120
##                (intercept)    0.031     0.025  -0.017 0.080   0.207
##  Penalized robust MR-Egger   -0.600     0.386  -1.356 0.156   0.120
##                (intercept)    0.031     0.025  -0.017 0.080   0.207
```

```r
### remove eighth SNP
bx8 <- c(0.0583,0.0677,0.0488,0.0488,0.0862,0.0488,0.0770,0.0770)
bxse8 <- c(0.0096,0.0119,0.0097,0.0097,0.0163,0.0097,0.0142,0.0094)

by8 <- c(0.0047,0.0093,-0.0142,-0.0071,-0.0392,0.0158,-0.0055,-0.0252)
byse8 <- c(0.0184,0.022,0.0176,0.0171,0.0347,0.0169,0.0195,0.0159)



MRInputObject8 <- mr_input(bx = bx8,
                           bxse = bxse8,
                           by = by8,
                           byse = byse8)

mr_allmethods(MRInputObject8, method = "all")
```

```
##                     Method Estimate Std Error 95% CI       P-value
##              Simple median   -0.108     0.140  -0.382 0.165   0.438
##            Weighted median   -0.126     0.139  -0.398 0.146   0.362
##  Penalized weighted median   -0.126     0.139  -0.398 0.146   0.362
##
##                        IVW   -0.115     0.106  -0.323 0.093   0.279
##              Penalized IVW   -0.115     0.106  -0.323 0.093   0.279
##                 Robust IVW   -0.116     0.089  -0.291 0.059   0.194
##       Penalized robust IVW   -0.116     0.089  -0.291 0.059   0.194
##
##                   MR-Egger   -0.607     0.511  -1.608 0.394   0.235
##                (intercept)    0.032     0.032  -0.031 0.094   0.325
##         Penalized MR-Egger   -0.607     0.511  -1.608 0.394   0.235
##                (intercept)    0.032     0.032  -0.031 0.094   0.325
##            Robust MR-Egger   -0.610     0.364  -1.323 0.103   0.094
##                (intercept)    0.032     0.024  -0.015 0.079   0.185
##  Penalized robust MR-Egger   -0.610     0.364  -1.323 0.103   0.094
##                (intercept)    0.032     0.024  -0.015 0.079   0.185
```

```r
### remove ninth SNP
bx9 <- c(0.0583,0.0677,0.0488,0.0488,0.0862,0.0488,0.0770,0.0770)
bxse9 <- c(0.0096,0.0119,0.0097,0.0097,0.0163,0.0097,0.0142,0.0142)

by9 <- c(0.0047,0.0093,-0.0142,-0.0071,-0.0392,0.0158,-0.0055,-0.0003)
byse9 <- c(0.0184,0.022,0.0176,0.0171,0.0347,0.0169,0.0195,0.0231)



MRInputObject9 <- mr_input(bx = bx9,
                           bxse = bxse9,
                           by = by9,
                           byse = byse9)

mr_allmethods(MRInputObject9, method = "all")
```

```
##                     Method Estimate Std Error 95% CI        P-value
##              Simple median   -0.038     0.142  -0.317 0.241   0.791
##            Weighted median   -0.029     0.146  -0.315 0.257   0.845
##  Penalized weighted median   -0.029     0.146  -0.315 0.257   0.845
##
##                        IVW   -0.034     0.114  -0.258 0.190   0.768
##              Penalized IVW   -0.034     0.114  -0.258 0.190   0.768
##                 Robust IVW   -0.033     0.081  -0.193 0.126   0.682
##       Penalized robust IVW   -0.033     0.081  -0.193 0.126   0.682
##
##                   MR-Egger   -0.252     0.553  -1.335 0.831   0.649
##                (intercept)    0.014     0.034  -0.053 0.080   0.687
##         Penalized MR-Egger   -0.252     0.553  -1.335 0.831   0.649
##                (intercept)    0.014     0.034  -0.053 0.080   0.687
##            Robust MR-Egger   -0.238     0.406  -1.034 0.558   0.558
##                (intercept)    0.013     0.027  -0.040 0.066   0.635
##  Penalized robust MR-Egger   -0.238     0.406  -1.034 0.558   0.558
##                (intercept)    0.013     0.027  -0.040 0.066   0.635
```

```
##library R package
library(TwoSampleMR)
```

```
## Welcome to TwoSampleMR.
## [>] Full documentation: https://mrcieu.github.io/TwoSampleMR
## [>] Check news(package='TwoSampleMR') for bug fixes and updates
## [>] By generating access tokens to retrieve data from the MR-Base
##     database you consent to having your email address logged on
##     our servers. For info on how this is used see logging_info()
## [>] NOTE: We will be rolling out extensive changes to the database
##     in the next few weeks. To ensure backwards compatibility please
##     keep the R package updated.
```

```
##
## Warning:
## You are running an old version of the TwoSampleMR package.
## This version:   0.4.26
## Latest version: 0.5.2
## Please consider updating using devtools::install_github('MRCIEU/TwoSampleMR')
```

```
##
## Attaching package: 'TwoSampleMR'
```

```
## The following objects are masked from 'package:MendelianRandomization':
##
##     mr_ivw, mr_median
```

```
### read exposure data (9 SNPs associated with AIS stroke)
AIS_stroke_dat <- read_exposure_data("C:/Users/12601/Desktop/MR_modifition/TwoSampleMR_exposure
AIS and AD.txt")

###print exposure data
AIS_stroke_dat
```

```
##          SNP beta.exposure se.exposure effect_allele.exposure
## 1  rs6825454        0.0583      0.0096                      C
## 2 rs11957829        0.0677      0.0119                      A
## 3  rs7304841        0.0488      0.0097                      A
## 4  rs4932370        0.0488      0.0097                      A
## 5 rs11867415        0.0862      0.0163                      G
## 6  rs2229383        0.0488      0.0097                      T
## 7   rs635634        0.0770      0.0142                      T
## 8  rs2005108        0.0770      0.0142                      T
## 9  rs3184504        0.0770      0.0094                      T
##   other_allele.exposure eaf.exposure pval.exposure gene.exposure
## 1                     T         0.31      7.43e-10           FGA
## 2                     G         0.82      7.51e-09  LOC100505841
## 3                     C         0.59      4.93e-08         PDE3A
## 4                     G         0.33      2.88e-08     FURIN-FES
## 5                     A         0.18      4.81e-08         PRPF8
## 6                     G         0.65      4.72e-08  ILF3-SLC44A2
## 7                     C         0.19      9.18e-09           ABO
## 8                     C         0.12      3.33e-08         MMP12
## 9                     C         0.45      2.17e-14         SH2B3
##   samplesize.exposure   exposure mr_keep.exposure pval_origin.exposure
## 1              514791 AIS_stroke             TRUE             reported
## 2              514791 AIS_stroke             TRUE             reported
## 3              514791 AIS_stroke             TRUE             reported
## 4              514791 AIS_stroke             TRUE             reported
## 5              514791 AIS_stroke             TRUE             reported
## 6              514791 AIS_stroke             TRUE             reported
## 7              440328 AIS_stroke             TRUE             reported
## 8              514791 AIS_stroke             TRUE             reported
## 9              514791 AIS_stroke             TRUE             reported
##   id.exposure data_source.exposure
## 1      gADkvv             textfile
## 2      gADkvv             textfile
## 3      gADkvv             textfile
## 4      gADkvv             textfile
## 5      gADkvv             textfile
## 6      gADkvv             textfile
## 7      gADkvv             textfile
## 8      gADkvv             textfile
## 9      gADkvv             textfile
```

```
### read outcome data (9 SNPs from AD GWAS)
AD_outcome_dat <- read_outcome_data(snps = AIS_stroke_dat$SNP,
                                    filename = "C:/Users/12601/Desktop/MR_modifition/TwoSampleM
R_outcome AIS and AD.csv",
                                    sep = ",",snp_col = "SNP",beta_col = "beta",se_col = "se",
                                    effect_allele_col = "effect_allele",other_allele_col = "oth
er_allele",
                                    gene_col = "gene",samplesize_col = "samplesize")
```

```
## Warning in format_data(as.data.frame(outcome_dat), type = "outcome", snps = snps, : The foll
owing columns are not present but are helpful for harmonisation
## eaf
```

```
###print outcome data
AD_outcome_dat
```

```
##          SNP beta.outcome se.outcome effect_allele.outcome other_allele.outcome
## 1  rs6825454       0.0047     0.0184                     C                    T
## 2 rs11957829       0.0093     0.0220                     A                    G
## 3  rs7304841      -0.0142     0.0176                     A                    C
## 4  rs4932370      -0.0071     0.0171                     A                    G
## 5 rs11867415      -0.0392     0.0347                     G                    A
## 6  rs2229383       0.0158     0.0169                     T                    G
## 7   rs635634      -0.0055     0.0195                     T                    C
## 8  rs2005108      -0.0003     0.0231                     T                    C
## 9  rs3184504      -0.0252     0.0159                     T                    C
##   pval.outcome gene.outcome samplesize.outcome outcome mr_keep.outcome
## 1       0.7961          FGA              54162      AD            TRUE
## 2       0.6732 LOC100505841              54162      AD            TRUE
## 3       0.4183        PDE3A              54162      AD            TRUE
## 4       0.6793    FURIN-FES              54162      AD            TRUE
## 5       0.2580        PRPF8              54162      AD            TRUE
## 6       0.3508 ILF3-SLC44A2              54162      AD            TRUE
## 7       0.7793          ABO              54162      AD            TRUE
## 8       0.9888        MMP12              54162      AD            TRUE
## 9       0.1134        SH2B3              54162      AD            TRUE
##   pval_origin.outcome id.outcome eaf.outcome data_source.outcome
## 1            reported     wNVJRi          NA            textfile
## 2            reported     wNVJRi          NA            textfile
## 3            reported     wNVJRi          NA            textfile
## 4            reported     wNVJRi          NA            textfile
## 5            reported     wNVJRi          NA            textfile
## 6            reported     wNVJRi          NA            textfile
## 7            reported     wNVJRi          NA            textfile
## 8            reported     wNVJRi          NA            textfile
## 9            reported     wNVJRi          NA            textfile
```

```
### harmonise exposure data and outcome data
dat <- harmonise_data(AIS_stroke_dat, AD_outcome_dat)
```

```
## Harmonising AIS_stroke (gADkvv) and AD (wNVJRi)
```

```
### set up unit for the exposure
dat$units.exposure <- "OR"

### set up unit for the outcome
dat$units.outcome <- "OR"
class(dat)
```

```
## [1] "data.frame"
```

```
### run Steiger filtering for each SNP
dat2 <- steiger_filtering(dat)
```

```
## Estimating correlation for quantitative trait.
```

```
## This method is an approximation, and may be numerically unstable.
```

```
## Ideally you should estimate r directly from independent replication samples.
```

```
## Use get_r_from_lor for binary traits.
```

```
## Estimating correlation for quantitative trait.
```

```
## This method is an approximation, and may be numerically unstable.
```

```
## Ideally you should estimate r directly from independent replication samples.
```

```
## Use get_r_from_lor for binary traits.
```

```
### MR analysis excluding instruments with the wrong direction of effects
mr_results <- mr(subset(dat2,steiger_dir))
```

```
## Analysing 'gADkvv' on 'wNVJRi'
```

```
### print mr_results
mr_results
```

```
##   id.exposure id.outcome outcome   exposure                    method nsnp
## 1      gADkvv     wNVJRi      AD AIS_stroke                  MR Egger    9
## 2      gADkvv     wNVJRi      AD AIS_stroke           Weighted median    9
## 3      gADkvv     wNVJRi      AD AIS_stroke Inverse variance weighted    9
## 4      gADkvv     wNVJRi      AD AIS_stroke               Simple mode    9
## 5      gADkvv     wNVJRi      AD AIS_stroke             Weighted mode    9
##             b         se      pval
## 1 -0.51240782 0.48462178 0.3254762
## 2 -0.08860460 0.12893130 0.4919420
## 3 -0.10250231 0.09999699 0.3053377
## 4 -0.03497278 0.20605605 0.8694401
## 5 -0.07031192 0.18137950 0.7083847
```
