# Supplementary material for: Stroke and Alzheimer’s Disease: A Mendelian Randomization Study
Source: Front Genet. 2020 Jul 14;11:581. doi: 10.3389/fgene.2020.00581 (PMC7371994; doi:10.3389/fgene.2020.00581)
Supplement: Supplementary file 3 [file Data_Sheet_3.PDF]

Supplementary-File-3-LAS_stroke-and-AD.R

12601

2020-03-27

```
###library packages
library(MendelianRandomization)
```

```
## Warning: package 'MendelianRandomization' was built under R version 3.5.3
```

```
### all 3 SNPs(rs10820405, rs12124533, rs2107595)
bx <- c(0.1823,0.1570,0.1906)
bxse <- c(0.0341,0.0262,0.0233)

by <- c(-0.0086,0.0321,-0.0038)
byse <- c(0.0198,0.0187,0.0211)
### create MRInputObject
MRInputObject <- mr_input(bx = bx,
                          bxse = bxse,
                          by = by,
                          byse = byse)
### output the results for all methods
mr_allmethods(MRInputObject, method = "all")
```

```
##                     Method Estimate Std Error 95% CI        P-value
##              Simple median   -0.020     0.087  -0.191  0.151   0.819
##            Weighted median   -0.022     0.085  -0.189  0.144   0.793
##  Penalized weighted median   -0.022     0.085  -0.189  0.144   0.793
##
##                        IVW    0.037     0.065  -0.090  0.164   0.568
##              Penalized IVW    0.037     0.065  -0.090  0.164   0.568
##                 Robust IVW    0.034     0.053  -0.070  0.137   0.523
##       Penalized robust IVW    0.034     0.053  -0.070  0.137   0.523
##
##                   MR-Egger   -1.220     0.788  -2.766  0.325   0.122
##                (intercept)    0.222     0.139  -0.050  0.494   0.110
##         Penalized MR-Egger   -1.220     0.788  -2.766  0.325   0.122
##                (intercept)    0.222     0.139  -0.050  0.494   0.110
##            Robust MR-Egger   -1.220     0.234  -1.679 -0.762   0.000
##                (intercept)    0.222     0.037   0.149  0.295   0.000
##  Penalized robust MR-Egger   -1.220     0.234  -1.679 -0.762   0.000
##                (intercept)    0.222     0.037   0.149  0.295   0.000
```

```
### output the results for ivw methods, including Heterogeneity test
mr_ivw(MRInputObject)
```

```
##
## Inverse-variance weighted method
## (variants uncorrelated, fixed-effect model)
##
## Number of Variants : 3
##
## ------------------------------------------------------------------
##  Method Estimate Std Error  95% CI       p-value
##     IVW    0.037     0.065 -0.090, 0.164   0.568
## ------------------------------------------------------------------
## Residual standard error =  1.192
## Residual standard error is set to 1 in calculation of confidence interval by fixed-effect as
sumption.
## Heterogeneity test statistic = 2.8418 on 2 degrees of freedom, (p-value = 0.2415)
```

```
#### first SNP (rs10820405)
bx1 <- c(0.1823)
bxse1 <- c(0.0341)

by1 <- c(-0.0086)
byse1 <- c(0.0198)

###creat MRInputObject1
MRInputObject1 <- mr_input(bx = bx1,
                          bxse = bxse1,
                          by = by1,
                          byse = byse1)
### output the results for ivw method
mr_ivw(MRInputObject1)
```

```
##
## Inverse-variance weighted method
## (variants uncorrelated, fixed-effect model)
##
## Number of Variants : 1
##
## ------------------------------------------------------------------
##  Method Estimate Std Error  95% CI       p-value
##     IVW   -0.047     0.109 -0.260, 0.166   0.664
## ------------------------------------------------------------------
## Residual standard error =  1.000
## Residual standard error is set to 1 in calculation of confidence interval by fixed-effect as
sumption.
## Heterogeneity is not calculated when weights are penalized, or when there is only one varian
t in the analysis.
```

```r
#### second SNP (rs12124533)
bx2 <- c(0.1570)
bxse2 <- c(0.0262)

by2 <- c(0.0321)
byse2 <- c(0.0187)

###creat MRInputObject2
MRInputObject2 <- mr_input(bx = bx2,
                           bxse = bxse2,
                           by = by2,
                           byse = byse2)
### output the results for ivw method
mr_ivw(MRInputObject2)
```

```
##
## Inverse-variance weighted method
## (variants uncorrelated, fixed-effect model)
##
## Number of Variants : 1
##
## ------------------------------------------------------------------
##  Method Estimate Std Error  95% CI       p-value
##     IVW    0.204     0.119 -0.029, 0.438   0.086
## ------------------------------------------------------------------
## Residual standard error =  1.000
## Residual standard error is set to 1 in calculation of confidence interval by fixed-effect as
sumption.
## Heterogeneity is not calculated when weights are penalized, or when there is only one varian
t in the analysis.
```

```r
#### third SNP (rs2107595)
bx3 <- c(0.1906)
bxse3 <- c(0.0233)

by3 <- c(-0.0038)
byse3 <- c(0.0211)

###creat MRInputObject3
MRInputObject3 <- mr_input(bx = bx3,
                           bxse = bxse3,
                           by = by3,
                           byse = byse3)
### output the results for ivw method
mr_ivw(MRInputObject3)
```

```
##
## Inverse-variance weighted method
## (variants uncorrelated, fixed-effect model)
##
## Number of Variants : 1
##
## ------------------------------------------------------------------
##  Method Estimate Std Error  95% CI       p-value
##     IVW   -0.020     0.111 -0.237, 0.197   0.857
## ------------------------------------------------------------------
## Residual standard error =  1.000
## Residual standard error is set to 1 in calculation of confidence interval by fixed-effect as
sumption.
## Heterogeneity is not calculated when weights are penalized, or when there is only one varian
t in the analysis.
```

```
##library R package
library(TwoSampleMR)
```

```
## Welcome to TwoSampleMR.
## [>] Full documentation: https://mrcieu.github.io/TwoSampleMR
## [>] Check news(package='TwoSampleMR') for bug fixes and updates
## [>] By generating access tokens to retrieve data from the MR-Base
##     database you consent to having your email address logged on
##     our servers. For info on how this is used see logging_info()
## [>] NOTE: We will be rolling out extensive changes to the database
##     in the next few weeks. To ensure backwards compatibility please
##     keep the R package updated.
```

```
##
## Warning:
## You are running an old version of the TwoSampleMR package.
## This version:   0.4.26
## Latest version: 0.5.2
## Please consider updating using devtools::install_github('MRCIEU/TwoSampleMR')
```

```
##
## Attaching package: 'TwoSampleMR'
```

```
## The following objects are masked from 'package:MendelianRandomization':
##
##     mr_ivw, mr_median
```

```
### read exposure data (3 SNPs associated with LAS stroke)
LAS_stroke_dat <- read_exposure_data("C:/Users/12601/Desktop/MR_modifition/TwoSampleMR_exposure
LAS and AD.txt")

###print exposure data
LAS_stroke_dat
```

```
##          SNP beta.exposure se.exposure effect_allele.exposure
## 1 rs10820405        0.1823      0.0341                      G
## 2 rs12124533        0.1570      0.0262                      T
## 3  rs2107595        0.1906      0.0233                      A
##   other_allele.exposure eaf.exposure pval.exposure gene.exposure
## 1                     A         0.82      4.51e-08     LINC01492
## 2                     C         0.24      1.22e-08        TSPAN2
## 3                     G         0.24      3.65e-15  HDAC9 - TWIST1
##   samplesize.exposure   exposure mr_keep.exposure pval_origin.exposure
## 1              301663 LAS_stroke             TRUE             reported
## 2              352317 LAS_stroke             TRUE             reported
## 3              352317 LAS_stroke             TRUE             reported
##   id.exposure data_source.exposure
## 1      TQ217I             textfile
## 2      TQ217I             textfile
## 3      TQ217I             textfile
```

```
### read outcome data (3 SNPs from AD GWAS)
AD_outcome_dat <- read_outcome_data(snps = LAS_stroke_dat$SNP,
                                    filename = "C:/Users/12601/Desktop/MR_modifition/TwoSampleM
R_outcome LAS and AD.csv",
                                    sep = ",",snp_col = "SNP",beta_col = "beta",se_col = "se",
                                    effect_allele_col = "effect_allele",other_allele_col = "oth
er_allele",
                                    gene_col = "gene",samplesize_col = "samplesize")
```

```
## Warning in format_data(as.data.frame(outcome_dat), type = "outcome", snps = snps, : The foll
owing columns are not present but are helpful for harmonisation
## eaf
```

```
###print outcome data
AD_outcome_dat
```

```
##          SNP beta.outcome se.outcome effect_allele.outcome other_allele.outcome
## 1 rs10820405      -0.0086     0.0198                     G                    A
## 2 rs12124533       0.0321     0.0187                     T                    C
## 3  rs2107595      -0.0038     0.0211                     A                    G
##   pval.outcome gene.outcome samplesize.outcome outcome mr_keep.outcome
## 1      0.66440    LINC01492              54162      AD            TRUE
## 2      0.08658       TSPAN2              54162      AD            TRUE
## 3      0.85820 HDAC9 - TWIST1              54162      AD            TRUE
##   pval_origin.outcome id.outcome eaf.outcome data_source.outcome
## 1            reported     yfqxkd          NA            textfile
## 2            reported     yfqxkd          NA            textfile
## 3            reported     yfqxkd          NA            textfile
```

```
### harmonise exposure data and outcome data
dat <- harmonise_data(LAS_stroke_dat, AD_outcome_dat)
```

```
## Harmonising LAS_stroke (TQ217I) and AD (yfqxkd)
```

```
### set up unit for the exposure
dat$units.exposure <- "OR"

### set up unit for the outcome
dat$units.outcome <- "OR"
class(dat)
```

```
## [1] "data.frame"
```

```
### run Steiger filtering for each SNP
dat2 <- steiger_filtering(dat)
```

```
## Estimating correlation for quantitative trait.
```

```
## This method is an approximation, and may be numerically unstable.
```

```
## Ideally you should estimate r directly from independent replication samples.
```

```
## Use get_r_from_lor for binary traits.
```

```
## Estimating correlation for quantitative trait.
```

```
## This method is an approximation, and may be numerically unstable.
```

```
## Ideally you should estimate r directly from independent replication samples.
```

```
## Use get_r_from_lor for binary traits.
```

```
### MR analysis excluding instruments with the wrong direction of effects
mr_results <- mr(subset(dat2,steiger_dir))
```

```
## Analysing 'TQ2l7I' on 'yfqxkd'
```

```
### print mr_results
mr_results
```

```
##   id.exposure id.outcome outcome   exposure                    method nsnp
## 1      TQ2l7I     yfqxkd      AD LAS_stroke                  MR Egger    3
## 2      TQ2l7I     yfqxkd      AD LAS_stroke           Weighted median    3
## 3      TQ2l7I     yfqxkd      AD LAS_stroke Inverse variance weighted    3
## 4      TQ2l7I     yfqxkd      AD LAS_stroke               Simple mode    3
## 5      TQ2l7I     yfqxkd      AD LAS_stroke             Weighted mode    3
##             b         se      pval
## 1 -1.22049506 0.78848778 0.3651561
## 2 -0.02307069 0.08693200 0.7907108
## 3  0.03709439 0.07745333 0.6319915
## 4 -0.03366593 0.10808378 0.7849057
## 5 -0.03366593 0.11029951 0.7890324
```
