# Supplementary material for: Stroke and Alzheimer’s Disease: A Mendelian Randomization Study
Source: Front Genet. 2020 Jul 14;11:581. doi: 10.3389/fgene.2020.00581 (PMC7371994; doi:10.3389/fgene.2020.00581)
Supplement: Supplementary file 4 [file Data_Sheet_4.PDF]

Supplementary-File-4-CES_stroke-and-AD.R

12601

2020-03-27

```
###library packages
library(MendelianRandomization)
```

```
## Warning: package 'MendelianRandomization' was built under R version 3.5.3
```

```
### all 3 SNPs (rs6891174,rs13143308,rs12932445)
bx <- c(0.1044,0.2776,0.1823)
bxse <- c(0.0206,0.0193,0.0213)

by <- c(-0.0021,-0.0128,-0.0261)
byse <- c(0.017,0.0188,0.0216)
### create MRInputObject
MRInputObject <- mr_input(bx = bx,
                          bxse = bxse,
                          by = by,
                          byse = byse)
### output the results for all methods
mr_allmethods(MRInputObject, method = "all")
```

```
##                    Method Estimate Std Error 95% CI        P-value
##             Simple median   -0.046     0.076  -0.195 0.102   0.543
##           Weighted median   -0.057     0.059  -0.173 0.058   0.331
##  Penalized weighted median   -0.057     0.059  -0.173 0.058   0.331
##
##                       IVW   -0.064     0.055  -0.173 0.044   0.245
##             Penalized IVW   -0.064     0.055  -0.173 0.044   0.245
##                Robust IVW   -0.063     0.036  -0.133 0.007   0.078
##      Penalized robust IVW   -0.063     0.036  -0.133 0.007   0.078
##
##                  MR-Egger   -0.062     0.146  -0.349 0.225   0.673
##               (intercept)   -0.001     0.029  -0.057 0.056   0.985
##        Penalized MR-Egger   -0.062     0.146  -0.349 0.225   0.673
##               (intercept)   -0.001     0.029  -0.057 0.056   0.985
##           Robust MR-Egger   -0.062     0.039  -0.139 0.016   0.117
##               (intercept)    0.000     0.009  -0.018 0.017   0.960
##  Penalized robust MR-Egger   -0.062     0.039  -0.139 0.016   0.117
##               (intercept)    0.000     0.009  -0.018 0.017   0.960
```

```
### output the results for ivw methods, including Heterogeneity test
mr_ivw(MRInputObject)
```

```
##
## Inverse-variance weighted method
## (variants uncorrelated, fixed-effect model)
##
## Number of Variants : 3
##
## ------------------------------------------------------------------
##  Method Estimate Std Error  95% CI       p-value
##     IVW   -0.064     0.055 -0.173, 0.044   0.245
## ------------------------------------------------------------------
## Residual standard error =  0.543
## Residual standard error is set to 1 in calculation of confidence interval by fixed-effect as
sumption.
## Residual standard error is set to 1 in calculation of confidence interval when its estimate
is less than 1.
## Heterogeneity test statistic = 0.5889 on 2 degrees of freedom, (p-value = 0.7450)
```

```
#### first SNP (rs6891174)
bx1 <- c(0.1044)
bxse1 <- c(0.0206)

by1 <- c(-0.0021)
byse1 <- c(0.017)

###creat MRInputObject1
MRInputObject1 <- mr_input(bx = bx1,
                           bxse = bxse1,
                           by = by1,
                           byse = byse1)
### output the results for ivw method
mr_ivw(MRInputObject1)
```

```
##
## Inverse-variance weighted method
## (variants uncorrelated, fixed-effect model)
##
## Number of Variants : 1
##
## ------------------------------------------------------------------
##  Method Estimate Std Error  95% CI       p-value
##     IVW   -0.020     0.163 -0.339, 0.299   0.902
## ------------------------------------------------------------------
## Residual standard error =  1.000
## Residual standard error is set to 1 in calculation of confidence interval by fixed-effect as
sumption.
## Heterogeneity is not calculated when weights are penalized, or when there is only one varian
t in the analysis.
```

```r
#### second SNP (rs13143308)
bx2 <- c(0.2776)
bxse2 <- c(0.0193)

by2 <- c(-0.0128)
byse2 <- c(0.0188)

###creat MRInputObject2
MRInputObject2 <- mr_input(bx = bx2,
                           bxse = bxse2,
                           by = by2,
                           byse = byse2)
### output the results for ivw method
mr_ivw(MRInputObject2)
```

```
##
## Inverse-variance weighted method
## (variants uncorrelated, fixed-effect model)
##
## Number of Variants : 1
##
## ------------------------------------------------------------------
##  Method Estimate Std Error  95% CI       p-value
##     IVW   -0.046     0.068 -0.179, 0.087   0.496
## ------------------------------------------------------------------
## Residual standard error =  1.000
## Residual standard error is set to 1 in calculation of confidence interval by fixed-effect as
sumption.
## Heterogeneity is not calculated when weights are penalized, or when there is only one varian
t in the analysis.
```

```r
#### third SNP (rs12932445)
bx3 <- c(0.1823)
bxse3 <- c(0.0213)

by3 <- c(-0.0261)
byse3 <- c(0.0216)

###creat MRInputObject3
MRInputObject3 <- mr_input(bx = bx3,
                           bxse = bxse3,
                           by = by3,
                           byse = byse3)
### output the results for ivw method
mr_ivw(MRInputObject3)
```

```
##
## Inverse-variance weighted method
## (variants uncorrelated, fixed-effect model)
##
## Number of Variants : 1
##
## ------------------------------------------------------------------
##  Method Estimate Std Error  95% CI       p-value
##     IVW   -0.143     0.118 -0.375, 0.089   0.227
## ------------------------------------------------------------------
## Residual standard error =  1.000
## Residual standard error is set to 1 in calculation of confidence interval by fixed-effect as
sumption.
## Heterogeneity is not calculated when weights are penalized, or when there is only one varian
t in the analysis.
```

```
##library R package
library(TwoSampleMR)
```

```
## Welcome to TwoSampleMR.
## [>] Full documentation: https://mrcieu.github.io/TwoSampleMR
## [>] Check news(package='TwoSampleMR') for bug fixes and updates
## [>] By generating access tokens to retrieve data from the MR-Base
##     database you consent to having your email address logged on
##     our servers. For info on how this is used see logging_info()
## [>] NOTE: We will be rolling out extensive changes to the database
##     in the next few weeks. To ensure backwards compatibility please
##     keep the R package updated.
```

```
##
## Warning:
## You are running an old version of the TwoSampleMR package.
## This version:   0.4.26
## Latest version: 0.5.2
## Please consider updating using devtools::install_github('MRCIEU/TwoSampleMR')
```

```
##
## Attaching package: 'TwoSampleMR'
```

```
## The following objects are masked from 'package:MendelianRandomization':
##
##     mr_ivw, mr_median
```

```
### read exposure data (3 SNPs associated with CES stroke)
CES_stroke_dat <- read_exposure_data("C:/Users/12601/Desktop/MR_modifition/TwoSampleMR_exposure
CES and AD.txt")

###print exposure data
CES_stroke_dat
```

```
##          SNP beta.exposure se.exposure effect_allele.exposure
## 1  rs6891174        0.1044      0.0206                      A
## 2 rs13143308        0.2776      0.0193                      T
## 3 rs12932445        0.1823      0.0213                      C
##   other_allele.exposure eaf.exposure pval.exposure gene.exposure
## 1                     G         0.35      5.82e-09        NKX2-5
## 2                     G         0.34      1.86e-47         PITX2
## 3                     T         0.21      6.86e-18         ZFHX3
##   samplesize.exposure   exposure mr_keep.exposure pval_origin.exposure
## 1              412813 CES_stroke             TRUE             reported
## 2              412813 CES_stroke             TRUE             reported
## 3              412813 CES_stroke             TRUE             reported
##   id.exposure data_source.exposure
## 1      TQ217I             textfile
## 2      TQ217I             textfile
## 3      TQ217I             textfile
```

```
### read outcome data (3 SNPs from AD GWAS)
AD_outcome_dat <- read_outcome_data(snps = CES_stroke_dat$SNP,
                                    filename = "C:/Users/12601/Desktop/MR_modifition/TwoSampleM
R_outcome CES and AD.csv",
                                    sep = ",",snp_col = "SNP",beta_col = "beta",se_col = "se",
                                    effect_allele_col = "effect_allele",other_allele_col = "oth
er_allele",
                                    gene_col = "gene",samplesize_col = "samplesize")
```

```
## Warning in format_data(as.data.frame(outcome_dat), type = "outcome", snps = snps, : The foll
owing columns are not present but are helpful for harmonisation
## eaf
```

```
###print outcome data
AD_outcome_dat
```

```
##          SNP beta.outcome se.outcome effect_allele.outcome other_allele.outcome
## 1  rs6891174      -0.0021     0.0170                     A                    G
## 2 rs13143308      -0.0128     0.0188                     T                    G
## 3 rs12932445      -0.0261     0.0216                     C                    T
##   pval.outcome gene.outcome samplesize.outcome outcome mr_keep.outcome
## 1       0.9006       NKX2-5              54162      AD            TRUE
## 2       0.4972        PITX2              54162      AD            TRUE
## 3       0.2272        ZFHX3              54162      AD            TRUE
##   pval_origin.outcome id.outcome eaf.outcome data_source.outcome
## 1            reported     yfqxkd          NA            textfile
## 2            reported     yfqxkd          NA            textfile
## 3            reported     yfqxkd          NA            textfile
```

```
### harmonise exposure data and outcome data
dat <- harmonise_data(CES_stroke_dat, AD_outcome_dat)
```

```
## Harmonising CES_stroke (TQ217I) and AD (yfqxkd)
```

```
### set up unit for the exposure
dat$units.exposure <- "OR"

### set up unit for the outcome
dat$units.outcome <- "OR"
class(dat)
```

```
## [1] "data.frame"
```

```
### run Steiger filtering for each SNP
dat2 <- steiger_filtering(dat)
```

```
## Estimating correlation for quantitative trait.
```

```
## This method is an approximation, and may be numerically unstable.
```

```
## Ideally you should estimate r directly from independent replication samples.
```

```
## Use get_r_from_lor for binary traits.
```

```
## Estimating correlation for quantitative trait.
```

```
## This method is an approximation, and may be numerically unstable.
```

```
## Ideally you should estimate r directly from independent replication samples.
```

```
## Use get_r_from_lor for binary traits.
```

```
### MR analysis excluding instruments with the wrong direction of effects
mr_results <- mr(subset(dat2,steiger_dir))
```

```
## Analysing 'TQ217I' on 'yfqxkd'
```

```
### print mr_results
mr_results
```

```
##   id.exposure id.outcome outcome   exposure                    method nsnp
## 1      TQ2l7I     yfqxkd      AD CES_stroke                  MR Egger    3
## 2      TQ2l7I     yfqxkd      AD CES_stroke           Weighted median    3
## 3      TQ2l7I     yfqxkd      AD CES_stroke Inverse variance weighted    3
## 4      TQ2l7I     yfqxkd      AD CES_stroke               Simple mode    3
## 5      TQ2l7I     yfqxkd      AD CES_stroke             Weighted mode    3
##             b         se      pval
## 1 -0.06176937 0.14634180 0.7457321
## 2 -0.05696674 0.05937966 0.3373751
## 3 -0.06425552 0.05530198 0.2452750
## 4 -0.03310068 0.09199862 0.7534406
## 5 -0.04332007 0.06629379 0.5805493
```
