# Supplementary material for: Stroke and Alzheimer’s Disease: A Mendelian Randomization Study
Source: Front Genet. 2020 Jul 14;11:581. doi: 10.3389/fgene.2020.00581 (PMC7371994; doi:10.3389/fgene.2020.00581)
Supplement: Supplementary file 5 [file Data_Sheet_5.PDF]

Supplementary-File-5-AD-and-any-stroke.R

12601

2020-04-25

```
###library packages
library(MendelianRandomization)
```

```
## Warning: package 'MendelianRandomization' was built under R version 3.5.3
```

```
print("##############------ MR analysis between AD and any stroke ------################")
```

```
## [1] "##############------ MR analysis between AD and any stroke ------################"
```

```
### all 12 SNPs (rs6656401, rs6733839, rs10948363, rs9271192, rs11771145, rs28834970, rs933189
6, rs11218343, rs10498633, rs8093731, rs4147929,rs3865444)
bx <- c(0.1567, 0.188, 0.0978, 0.1044, -0.1024, 0.0959, -0.1457, -0.2697, -0.1044,-0.6136,0.134
8,-0.0954)
bxse <- c(0.0202, 0.0176, 0.0177, 0.0206, 0.0167, 0.0162, 0.0175, 0.041, 0.0199, 0.1123, 0.0224
,0.0175)

by <- c(0.0075, -0.0075, 0.0034, 0.0006, 0.0152, -0.0014, 0.0002, 0.006, 0.0094, 0.0079, 0.016,
-0.0133)
byse <- c(0.0116, 0.0087, 0.0093, 0.0104, 0.0082, 0.0083, 0.0083, 0.0149, 0.0102, 0.0237, 0.010
3,0.0089)
### create MRInputObject
MRInputObject <- mr_input(bx = bx,
                          bxse = bxse,
                          by = by,
                          byse = byse)
### output the results for all methods
mr_allmethods(MRInputObject, method = "all")
```

```
##                      Method Estimate Std Error 95% CI        P-value
##               Simple median   -0.007     0.026  -0.059 0.044   0.786
##             Weighted median   -0.013     0.026  -0.064 0.037   0.604
##  Penalized weighted median   -0.013     0.026  -0.064 0.037   0.604
##
##                         IVW   -0.007     0.019  -0.044 0.030   0.708
##               Penalized IVW   -0.007     0.019  -0.044 0.030   0.708
##                  Robust IVW   -0.008     0.016  -0.040 0.024   0.614
##        Penalized robust IVW   -0.008     0.016  -0.040 0.024   0.614
##
##                    MR-Egger   -0.019     0.041  -0.099 0.061   0.639
##                 (intercept)    0.002     0.006  -0.010 0.014   0.738
##          Penalized MR-Egger   -0.019     0.041  -0.099 0.061   0.639
##                 (intercept)    0.002     0.006  -0.010 0.014   0.738
##             Robust MR-Egger   -0.021     0.018  -0.057 0.015   0.251
##                 (intercept)    0.002     0.005  -0.008 0.012   0.632
##  Penalized robust MR-Egger   -0.021     0.018  -0.057 0.015   0.251
##                 (intercept)    0.002     0.005  -0.008 0.012   0.632
```

```
print("#########------ sensitive analysis of 12 SNPs for AD and any stroke ------##########")
```

```
## [1] "#########------ sensitive analysis of 12 SNPs for AD and any stroke ------##########"
```

```
### remove first SNP (rs6656401)

bx1 <- c( 0.188, 0.0978, 0.1044, -0.1024, 0.0959, -0.1457, -0.2697, -0.1044,-0.6136,0.1348,-0.0954)
bxse1 <- c(0.0176, 0.0177, 0.0206, 0.0167, 0.0162, 0.0175, 0.041, 0.0199, 0.1123, 0.0224,0.0175)

by1 <- c( -0.0075, 0.0034, 0.0006, 0.0152, -0.0014, 0.0002, 0.006, 0.0094, 0.0079, 0.016,-0.0133)
byse1 <- c( 0.0087, 0.0093, 0.0104, 0.0082, 0.0083, 0.0083, 0.0149, 0.0102, 0.0237, 0.0103,0.0089)
### create MRInputObject
MRInputObject1 <- mr_input(bx = bx1,
                          bxse = bxse1,
                          by = by1,
                          byse = byse1)
### output the results for all methods
mr_allmethods(MRInputObject1, method = "all")
```

```
##                     Method Estimate Std Error 95% CI        P-value
##              Simple median   -0.013     0.028  -0.068 0.042   0.645
##            Weighted median   -0.014     0.026  -0.064 0.036   0.593
##  Penalized weighted median   -0.014     0.026  -0.064 0.036   0.593
##                                                                    
##                        IVW   -0.011     0.019  -0.049 0.027   0.578
##              Penalized IVW   -0.011     0.019  -0.049 0.027   0.578
##                 Robust IVW   -0.013     0.017  -0.047 0.021   0.448
##       Penalized robust IVW   -0.013     0.017  -0.047 0.021   0.448
##                                                                    
##                   MR-Egger   -0.021     0.042  -0.103 0.060   0.607
##                (intercept)    0.002     0.006  -0.011 0.014   0.770
##         Penalized MR-Egger   -0.021     0.042  -0.103 0.060   0.607
##                (intercept)    0.002     0.006  -0.011 0.014   0.770
##            Robust MR-Egger   -0.023     0.019  -0.061 0.015   0.238
##                (intercept)    0.002     0.006  -0.009 0.013   0.723
##  Penalized robust MR-Egger   -0.023     0.019  -0.061 0.015   0.238
##                (intercept)    0.002     0.006  -0.009 0.013   0.723
```

```
### remove second SNP (rs6733839)
bx2 <- c(0.1567, 0.0978, 0.1044, -0.1024, 0.0959, -0.1457, -0.2697, -0.1044,-0.6136,0.1348,-0.0
954)
bxse2 <- c(0.0202, 0.0177, 0.0206, 0.0167, 0.0162, 0.0175, 0.041, 0.0199, 0.1123, 0.0224,0.0175
)

by2 <- c(0.0075, 0.0034, 0.0006, 0.0152, -0.0014, 0.0002, 0.006, 0.0094, 0.0079, 0.016,-0.0133)
byse2 <- c(0.0116, 0.0093, 0.0104, 0.0082, 0.0083, 0.0083, 0.0149, 0.0102, 0.0237, 0.0103,0.008
9)
### create MRInputObject
MRInputObject2 <- mr_input(bx = bx2,
                          bxse = bxse2,
                          by = by2,
                          byse = byse2)
### output the results for all methods
mr_allmethods(MRInputObject2, method = "all")
```

```
##                     Method Estimate Std Error 95% CI        P-value
##              Simple median   -0.001     0.030  -0.059 0.057   0.963
##            Weighted median   -0.010     0.028  -0.064 0.045   0.725
##  Penalized weighted median   -0.010     0.028  -0.064 0.045   0.725
##                                                                    
##                        IVW   -0.001     0.020  -0.041 0.040   0.978
##              Penalized IVW   -0.001     0.020  -0.041 0.040   0.978
##                 Robust IVW   -0.001     0.018  -0.036 0.035   0.963
##       Penalized robust IVW   -0.001     0.018  -0.036 0.035   0.963
##                                                                    
##                   MR-Egger   -0.011     0.043  -0.095 0.074   0.801
##                (intercept)    0.002     0.006  -0.011 0.014   0.784
##         Penalized MR-Egger   -0.011     0.043  -0.095 0.074   0.801
##                (intercept)    0.002     0.006  -0.011 0.014   0.784
##            Robust MR-Egger   -0.014     0.017  -0.046 0.019   0.412
##                (intercept)    0.002     0.005  -0.008 0.012   0.640
##  Penalized robust MR-Egger   -0.014     0.017  -0.046 0.019   0.412
##                (intercept)    0.002     0.005  -0.008 0.012   0.640
```

```r
### remove third SNP (rs10948363)
bx3 <- c(0.1567, 0.188, 0.1044, -0.1024, 0.0959, -0.1457, -0.2697, -0.1044,-0.6136,0.1348,-0.09
54)
bxse3 <- c(0.0202, 0.0176, 0.0206, 0.0167, 0.0162, 0.0175, 0.041, 0.0199, 0.1123, 0.0224,0.0175
)

by3 <- c(0.0075, -0.0075, 0.0006, 0.0152, -0.0014, 0.0002, 0.006, 0.0094, 0.0079, 0.016,-0.0133
)
byse3 <- c(0.0116, 0.0087, 0.0104, 0.0082, 0.0083, 0.0083, 0.0149, 0.0102, 0.0237, 0.0103,0.008
9)
### create MRInputObject
MRInputObject3 <- mr_input(bx = bx3,
                          bxse = bxse3,
                          by = by3,
                          byse = byse3)
### output the results for all methods
mr_allmethods(MRInputObject3, method = "all")
```

```
##                     Method Estimate Std Error 95% CI        P-value
##              Simple median   -0.013     0.027  -0.066 0.040   0.634
##            Weighted median   -0.014     0.025  -0.063 0.036   0.594
##  Penalized weighted median   -0.014     0.025  -0.063 0.036   0.594
##
##                        IVW   -0.009     0.019  -0.047 0.029   0.652
##              Penalized IVW   -0.009     0.019  -0.047 0.029   0.652
##                 Robust IVW   -0.010     0.016  -0.042 0.022   0.532
##       Penalized robust IVW   -0.010     0.016  -0.042 0.022   0.532
##
##                   MR-Egger   -0.017     0.043  -0.101 0.068   0.700
##                (intercept)    0.001     0.007  -0.012 0.015   0.836
##         Penalized MR-Egger   -0.017     0.043  -0.101 0.068   0.700
##                (intercept)    0.001     0.007  -0.012 0.015   0.836
##            Robust MR-Egger   -0.018     0.020  -0.057 0.021   0.376
##                (intercept)    0.002     0.006  -0.010 0.013   0.790
##  Penalized robust MR-Egger   -0.018     0.020  -0.057 0.021   0.376
##                (intercept)    0.002     0.006  -0.010 0.013   0.790
```

```r
### remove fourth SNP (rs9271192)
bx4 <- c(0.1567, 0.188, 0.0978, -0.1024, 0.0959, -0.1457, -0.2697, -0.1044,-0.6136,0.1348,-0.09
54)
bxse4 <- c(0.0202, 0.0176, 0.0177, 0.0167, 0.0162, 0.0175, 0.041, 0.0199, 0.1123, 0.0224,0.0175
)

by4 <- c(0.0075, -0.0075, 0.0034, 0.0152, -0.0014, 0.0002, 0.006, 0.0094, 0.0079, 0.016,-0.0133
)
byse4 <- c(0.0116, 0.0087, 0.0093, 0.0082, 0.0083, 0.0083, 0.0149, 0.0102, 0.0237, 0.0103,0.008
9)
### create MRInputObject
MRInputObject4 <- mr_input(bx = bx4,
                          bxse = bxse4,
                          by = by4,
                          byse = byse4)
### output the results for all methods
mr_allmethods(MRInputObject4, method = "all")
```

```
##                     Method Estimate Std Error 95% CI        P-value
##              Simple median   -0.013     0.027  -0.066 0.040   0.632
##            Weighted median   -0.014     0.025  -0.063 0.036   0.594
##  Penalized weighted median   -0.014     0.025  -0.063 0.036   0.594
##                                                                     
##                        IVW   -0.007     0.019  -0.046 0.031   0.700
##              Penalized IVW   -0.007     0.019  -0.046 0.031   0.700
##                 Robust IVW   -0.008     0.015  -0.038 0.021   0.572
##       Penalized robust IVW   -0.008     0.015  -0.038 0.021   0.572
##                                                                     
##                   MR-Egger   -0.019     0.043  -0.103 0.066   0.663
##                (intercept)    0.002     0.007  -0.011 0.015   0.766
##         Penalized MR-Egger   -0.019     0.043  -0.103 0.066   0.663
##                (intercept)    0.002     0.007  -0.011 0.015   0.766
##            Robust MR-Egger   -0.020     0.020  -0.059 0.018   0.303
##                (intercept)    0.002     0.006  -0.009 0.013   0.686
##  Penalized robust MR-Egger   -0.020     0.020  -0.059 0.018   0.303
##                (intercept)    0.002     0.006  -0.009 0.013   0.686
```

```
### remove fifth SNP (rs11771145)
bx5 <- c(0.1567, 0.188, 0.0978, 0.1044, 0.0959, -0.1457, -0.2697, -0.1044,-0.6136,0.1348,-0.095
4)
bxse5 <- c(0.0202, 0.0176, 0.0177, 0.0206, 0.0162, 0.0175, 0.041, 0.0199, 0.1123, 0.0224,0.0175
)

by5 <- c(0.0075, -0.0075, 0.0034, 0.0006, -0.0014, 0.0002, 0.006, 0.0094, 0.0079, 0.016,-0.0133
)
byse5 <- c(0.0116, 0.0087, 0.0093, 0.0104, 0.0083, 0.0083, 0.0149, 0.0102, 0.0237, 0.0103,0.008
9)
### create MRInputObject
MRInputObject5 <- mr_input(bx = bx5,
                          bxse = bxse5,
                          by = by5,
                          byse = byse5)
### output the results for all methods
mr_allmethods(MRInputObject5, method = "all")
```

```
##                     Method Estimate Std Error 95% CI        P-value
##              Simple median   -0.001     0.028  -0.056 0.053   0.960
##            Weighted median   -0.013     0.025  -0.062 0.037   0.607
##  Penalized weighted median   -0.013     0.025  -0.062 0.037   0.607
##                                                                     
##                        IVW    0.001     0.019  -0.037 0.039   0.952
##              Penalized IVW    0.001     0.019  -0.037 0.039   0.952
##                 Robust IVW   -0.003     0.020  -0.042 0.036   0.882
##       Penalized robust IVW   -0.003     0.020  -0.042 0.036   0.882
##                                                                     
##                   MR-Egger   -0.033     0.041  -0.112 0.047   0.421
##                (intercept)    0.006     0.006  -0.006 0.019   0.344
##         Penalized MR-Egger   -0.033     0.041  -0.112 0.047   0.421
##                (intercept)    0.006     0.006  -0.006 0.019   0.344
##            Robust MR-Egger   -0.031     0.021  -0.072 0.009   0.126
##                (intercept)    0.006     0.005  -0.004 0.016   0.271
##  Penalized robust MR-Egger   -0.031     0.021  -0.072 0.009   0.126
##                (intercept)    0.006     0.005  -0.004 0.016   0.271
```

```
### remove sixth SNP (rs28834970)
bx6 <- c(0.1567, 0.188, 0.0978, 0.1044, -0.1024, -0.1457, -0.2697, -0.1044,-0.6136,0.1348,-0.09
54)
bxse6 <- c(0.0202, 0.0176, 0.0177, 0.0206, 0.0167, 0.0175, 0.041, 0.0199, 0.1123, 0.0224,0.0175
)

by6 <- c(0.0075, -0.0075, 0.0034, 0.0006, 0.0152, 0.0002, 0.006, 0.0094, 0.0079, 0.016,-0.0133)
byse6 <- c(0.0116, 0.0087, 0.0093, 0.0104, 0.0082, 0.0083, 0.0149, 0.0102, 0.0237, 0.0103,0.008
9)
### create MRInputObject
MRInputObject6 <- mr_input(bx = bx6,
                           bxse = bxse6,
                           by = by6,
                           byse = byse6)
### output the results for all methods
mr_allmethods(MRInputObject6, method = "all")
```

```
##                     Method Estimate Std Error 95% CI        P-value
##              Simple median   -0.001     0.028  -0.055 0.053   0.960
##            Weighted median   -0.014     0.026  -0.064 0.037   0.597
##  Penalized weighted median   -0.014     0.026  -0.064 0.037   0.597
##
##                        IVW   -0.007     0.020  -0.045 0.032   0.734
##              Penalized IVW   -0.007     0.020  -0.045 0.032   0.734
##                 Robust IVW   -0.007     0.015  -0.037 0.022   0.619
##       Penalized robust IVW   -0.007     0.015  -0.037 0.022   0.619
##
##                   MR-Egger   -0.021     0.044  -0.106 0.065   0.633
##                (intercept)    0.003     0.007  -0.011 0.016   0.713
##         Penalized MR-Egger   -0.021     0.044  -0.106 0.065   0.633
##                (intercept)    0.003     0.007  -0.011 0.016   0.713
##            Robust MR-Egger   -0.023     0.022  -0.066 0.020   0.300
##                (intercept)    0.003     0.006  -0.009 0.015   0.631
##  Penalized robust MR-Egger   -0.023     0.022  -0.066 0.020   0.300
##                (intercept)    0.003     0.006  -0.009 0.015   0.631
```

```
### remove seventh SNP (rs9331896)
bx7 <- c(0.1567, 0.188, 0.0978, 0.1044, -0.1024, 0.0959, -0.2697, -0.1044,-0.6136,0.1348,-0.095
4)
bxse7 <- c(0.0202, 0.0176, 0.0177, 0.0206, 0.0167, 0.0162, 0.041, 0.0199, 0.1123, 0.0224,0.0175
)

by7 <- c(0.0075, -0.0075, 0.0034, 0.0006, 0.0152, -0.0014, 0.006, 0.0094, 0.0079, 0.016,-0.0133
)
byse7 <- c(0.0116, 0.0087, 0.0093, 0.0104, 0.0082, 0.0083, 0.0149, 0.0102, 0.0237, 0.0103,0.008
9)
### create MRInputObject
MRInputObject7 <- mr_input(bx = bx7,
                           bxse = bxse7,
                           by = by7,
                           byse = byse7)
### output the results for all methods
mr_allmethods(MRInputObject7, method = "all")
```

```
##                     Method Estimate Std Error 95% CI        P-value
##              Simple median   -0.013     0.029  -0.070 0.044   0.657
##            Weighted median   -0.014     0.027  -0.066 0.038   0.601
##  Penalized weighted median   -0.014     0.027  -0.066 0.038   0.601
##                                                                    
##                        IVW   -0.008     0.020  -0.047 0.032   0.703
##              Penalized IVW   -0.008     0.020  -0.047 0.032   0.703
##                 Robust IVW   -0.009     0.017  -0.041 0.024   0.593
##       Penalized robust IVW   -0.009     0.017  -0.041 0.024   0.593
##                                                                    
##                   MR-Egger   -0.019     0.043  -0.103 0.065   0.654
##                (intercept)    0.002     0.007  -0.011 0.015   0.758
##         Penalized MR-Egger   -0.019     0.043  -0.103 0.065   0.654
##                (intercept)    0.002     0.007  -0.011 0.015   0.758
##            Robust MR-Egger   -0.021     0.018  -0.055 0.014   0.247
##                (intercept)    0.002     0.005  -0.008 0.013   0.670
##  Penalized robust MR-Egger   -0.021     0.018  -0.055 0.014   0.247
##                (intercept)    0.002     0.005  -0.008 0.013   0.670
```

```
### remove eighth SNP (rs11218343)
bx8 <- c(0.1567, 0.188, 0.0978, 0.1044, -0.1024, 0.0959, -0.1457, -0.1044,-0.6136,0.1348,-0.095
4)
bxse8 <- c(0.0202, 0.0176, 0.0177, 0.0206, 0.0167, 0.0162, 0.0175, 0.0199, 0.1123, 0.0224,0.017
5)

by8 <- c(0.0075, -0.0075, 0.0034, 0.0006, 0.0152, -0.0014, 0.0002, 0.0094, 0.0079, 0.016,-0.013
3)
byse8 <- c(0.0116, 0.0087, 0.0093, 0.0104, 0.0082, 0.0083, 0.0083, 0.0102, 0.0237, 0.0103,0.008
9)
### create MRInputObject
MRInputObject8 <- mr_input(bx = bx8,
                          bxse = bxse8,
                          by = by8,
                          byse = byse8)
### output the results for all methods
mr_allmethods(MRInputObject8, method = "all")
```

```
##                     Method Estimate Std Error 95% CI        P-value
##              Simple median   -0.001     0.029  -0.059 0.056   0.963
##            Weighted median   -0.011     0.027  -0.064 0.041   0.673
##  Penalized weighted median   -0.011     0.027  -0.064 0.041   0.673
##                                                                    
##                        IVW   -0.005     0.020  -0.045 0.035   0.803
##              Penalized IVW   -0.005     0.020  -0.045 0.035   0.803
##                 Robust IVW   -0.006     0.016  -0.038 0.027   0.732
##       Penalized robust IVW   -0.006     0.016  -0.038 0.027   0.732
##                                                                    
##                   MR-Egger   -0.016     0.046  -0.106 0.075   0.735
##                (intercept)    0.002     0.007  -0.011 0.015   0.796
##         Penalized MR-Egger   -0.016     0.046  -0.106 0.075   0.735
##                (intercept)    0.002     0.007  -0.011 0.015   0.796
##            Robust MR-Egger   -0.017     0.018  -0.053 0.019   0.350
##                (intercept)    0.002     0.005  -0.008 0.012   0.678
##  Penalized robust MR-Egger   -0.017     0.018  -0.053 0.019   0.350
##                (intercept)    0.002     0.005  -0.008 0.012   0.678
```

```
### remove ninth SNP (rs10498633)
bx9 <- c(0.1567, 0.188, 0.0978, 0.1044, -0.1024, 0.0959, -0.1457, -0.2697, -0.6136,0.1348,-0.09
54)
bxse9 <- c(0.0202, 0.0176, 0.0177, 0.0206, 0.0167, 0.0162, 0.0175, 0.041, 0.1123, 0.0224,0.0175
)

by9 <- c(0.0075, -0.0075, 0.0034, 0.0006, 0.0152, -0.0014, 0.0002, 0.006, 0.0079, 0.016,-0.0133
)
byse9 <- c(0.0116, 0.0087, 0.0093, 0.0104, 0.0082, 0.0083, 0.0083, 0.0149, 0.0237, 0.0103,0.008
9)
### create MRInputObject
MRInputObject9 <- mr_input(bx = bx9,
                          bxse = bxse9,
                          by = by9,
                          byse = byse9)
### output the results for all methods
mr_allmethods(MRInputObject9, method = "all")
```

```
##                     Method Estimate Std Error 95% CI        P-value
##              Simple median   -0.001     0.028  -0.056 0.053   0.961
##            Weighted median   -0.013     0.025  -0.063 0.037   0.607
##  Penalized weighted median   -0.013     0.025  -0.063 0.037   0.607
##
##                        IVW   -0.004     0.019  -0.041 0.034   0.840
##              Penalized IVW   -0.004     0.019  -0.041 0.034   0.840
##                 Robust IVW   -0.005     0.018  -0.041 0.031   0.793
##       Penalized robust IVW   -0.005     0.018  -0.041 0.031   0.793
##
##                  MR-Egger   -0.024     0.041  -0.104 0.057   0.561
##                (intercept)    0.004     0.006  -0.009 0.016   0.580
##         Penalized MR-Egger   -0.024     0.041  -0.104 0.057   0.561
##                (intercept)    0.004     0.006  -0.009 0.016   0.580
##            Robust MR-Egger   -0.028     0.021  -0.069 0.013   0.185
##                (intercept)    0.005     0.006  -0.007 0.016   0.426
##  Penalized robust MR-Egger   -0.028     0.021  -0.069 0.013   0.185
##                (intercept)    0.005     0.006  -0.007 0.016   0.426
```

```
### remove tenth SNP (rs8093731)
bx10 <- c(0.1567, 0.188, 0.0978, 0.1044, -0.1024, 0.0959, -0.1457, -0.2697, -0.1044,0.1348,-0.0
954)
bxse10 <- c(0.0202, 0.0176, 0.0177, 0.0206, 0.0167, 0.0162, 0.0175, 0.041, 0.0199, 0.0224,0.017
5)

by10 <- c(0.0075, -0.0075, 0.0034, 0.0006, 0.0152, -0.0014, 0.0002, 0.006, 0.0094, 0.016,-0.013
3)
byse10 <- c(0.0116, 0.0087, 0.0093, 0.0104, 0.0082, 0.0083, 0.0083, 0.0149, 0.0102, 0.0103,0.00
89)
### create MRInputObject
MRInputObject10 <- mr_input(bx = bx10,
                          bxse = bxse10,
                          by = by10,
                          byse = byse10)
### output the results for all methods
mr_allmethods(MRInputObject10, method = "all")
```

```
##                     Method Estimate Std Error 95% CI        P-value
##              Simple median   -0.001     0.031  -0.062 0.059   0.965
##            Weighted median   -0.016     0.029  -0.073 0.041   0.590
##  Penalized weighted median   -0.016     0.029  -0.073 0.041   0.590
##
##                        IVW   -0.005     0.022  -0.048 0.038   0.811
##              Penalized IVW   -0.005     0.022  -0.048 0.038   0.811
##                 Robust IVW   -0.006     0.020  -0.044 0.032   0.762
##       Penalized robust IVW   -0.006     0.020  -0.044 0.032   0.762
##
##                   MR-Egger   -0.026     0.074  -0.171 0.119   0.723
##                (intercept)    0.003     0.010  -0.016 0.022   0.766
##         Penalized MR-Egger   -0.026     0.074  -0.171 0.119   0.723
##                (intercept)    0.003     0.010  -0.016 0.022   0.766
##            Robust MR-Egger   -0.031     0.047  -0.123 0.061   0.509
##                (intercept)    0.004     0.008  -0.012 0.020   0.651
##  Penalized robust MR-Egger   -0.031     0.047  -0.123 0.061   0.509
##                (intercept)    0.004     0.008  -0.012 0.020   0.651
```

```
### remove eleven SNP (rs4147929)
bx11 <- c(0.1567, 0.188, 0.0978, 0.1044, -0.1024, 0.0959, -0.1457, -0.2697, -0.1044,-0.6136,-0.0954)
bxse11 <- c(0.0202, 0.0176, 0.0177, 0.0206, 0.0167, 0.0162, 0.0175, 0.041, 0.0199, 0.1123,0.0175)

by11 <- c(0.0075, -0.0075, 0.0034, 0.0006, 0.0152, -0.0014, 0.0002, 0.006, 0.0094, 0.0079,-0.0133)
byse11 <- c(0.0116, 0.0087, 0.0093, 0.0104, 0.0082, 0.0083, 0.0083, 0.0149, 0.0102, 0.0237,0.0089)
### create MRInputObject
MRInputObject11 <- mr_input(bx = bx11,
                          bxse = bxse11,
                          by = by11,
                          byse = byse11)
### output the results for all methods
mr_allmethods(MRInputObject11, method = "all")
```

```
##                     Method Estimate Std Error 95% CI        P-value
##              Simple median   -0.013     0.027  -0.066 0.040   0.633
##            Weighted median   -0.014     0.025  -0.063 0.036   0.588
##  Penalized weighted median   -0.014     0.025  -0.063 0.036   0.588
##
##                        IVW   -0.015     0.019  -0.053 0.023   0.436
##              Penalized IVW   -0.015     0.019  -0.053 0.023   0.436
##                 Robust IVW   -0.015     0.015  -0.044 0.015   0.330
##       Penalized robust IVW   -0.015     0.015  -0.044 0.015   0.330
##
##                   MR-Egger   -0.019     0.040  -0.098 0.059   0.632
##                (intercept)    0.001     0.006  -0.011 0.013   0.907
##         Penalized MR-Egger   -0.019     0.040  -0.098 0.059   0.632
##                (intercept)    0.001     0.006  -0.011 0.013   0.907
##            Robust MR-Egger   -0.020     0.021  -0.061 0.022   0.347
##                (intercept)    0.001     0.006  -0.011 0.013   0.869
##  Penalized robust MR-Egger   -0.020     0.021  -0.061 0.022   0.347
##                (intercept)    0.001     0.006  -0.011 0.013   0.869
```

```r
### remove twelve SNP (rs3865444)
bx12 <- c(0.1567, 0.188, 0.0978, 0.1044, -0.1024, 0.0959, -0.1457, -0.2697, -0.1044,-0.6136,0.1
348)
bxse12 <- c(0.0202, 0.0176, 0.0177, 0.0206, 0.0167, 0.0162, 0.0175, 0.041, 0.0199, 0.1123, 0.02
24)

by12 <- c(0.0075, -0.0075, 0.0034, 0.0006, 0.0152, -0.0014, 0.0002, 0.006, 0.0094, 0.0079, 0.01
6)
byse12 <- c(0.0116, 0.0087, 0.0093, 0.0104, 0.0082, 0.0083, 0.0083, 0.0149, 0.0102, 0.0237, 0.0
103)
### create MRInputObject
MRInputObject12 <- mr_input(bx = bx12,
                          bxse = bxse12,
                          by = by12,
                          byse = byse12)
### output the results for all methods
mr_allmethods(MRInputObject12, method = "all")
```

```
##                    Method Estimate Std Error 95% CI        P-value
##             Simple median   -0.013     0.027  -0.065 0.040   0.631
##           Weighted median   -0.014     0.025  -0.063 0.036   0.590
##  Penalized weighted median   -0.014     0.025  -0.063 0.036   0.590
##
##                       IVW   -0.013     0.019  -0.051 0.024   0.491
##             Penalized IVW   -0.013     0.019  -0.051 0.024   0.491
##                Robust IVW   -0.014     0.016  -0.045 0.017   0.390
##      Penalized robust IVW   -0.014     0.016  -0.045 0.017   0.390
##
##                  MR-Egger   -0.007     0.041  -0.087 0.073   0.860
##               (intercept)   -0.001     0.006  -0.014 0.011   0.868
##        Penalized MR-Egger   -0.007     0.041  -0.087 0.073   0.860
##               (intercept)   -0.001     0.006  -0.014 0.011   0.868
##           Robust MR-Egger   -0.011     0.018  -0.047 0.025   0.553
##               (intercept)   -0.001     0.005  -0.011 0.010   0.921
##  Penalized robust MR-Egger   -0.011     0.018  -0.047 0.025   0.553
##               (intercept)   -0.001     0.005  -0.011 0.010   0.921
```

```r
print("##############------ MR analysis between AD and any stroke using Steiger filtering------
#################")
```

```
## [1] "##############------ MR analysis between AD and any stroke using Steiger filtering-----
-#################"
```

```r
##library R package
library(TwoSampleMR)
```

```
## Welcome to TwoSampleMR.
## [>] Full documentation: https://mrcieu.github.io/TwoSampleMR
## [>] Check news(package='TwoSampleMR') for bug fixes and updates
## [>] By generating access tokens to retrieve data from the MR-Base
##     database you consent to having your email address logged on
##     our servers. For info on how this is used see logging_info()
## [>] NOTE: We will be rolling out extensive changes to the database
##     in the next few weeks. To ensure backwards compatibility please
##     keep the R package updated.
```

```
##
## Warning:
## You are running an old version of the TwoSampleMR package.
## This version:   0.4.26
## Latest version: 0.5.3
## Please consider updating using devtools::install_github('MRCIEU/TwoSampleMR')
```

```
##
## Attaching package: 'TwoSampleMR'
```

```
## The following objects are masked from 'package:MendelianRandomization':
##
##     mr_ivw, mr_median
```

```
### read exposure data (12 SNPs associated with AD)
AD_exposure_dat <- read_exposure_data("C:/Users/12601/Desktop/MR_modifition/TwoSampleMR_exposur
e AD and AS.txt")

###print exposure data
AD_exposure_dat
```

```
##           SNP beta.exposure se.exposure effect_allele.exposure
## 1   rs6656401        0.1567      0.0202                      A
## 2   rs6733839        0.1880      0.0176                      T
## 3  rs10948363        0.0978      0.0177                      G
## 4   rs9271192        0.1044      0.0206                      C
## 5  rs11771145       -0.1024      0.0167                      A
## 6  rs28834970        0.0959      0.0162                      C
## 7   rs9331896       -0.1457      0.0175                      C
## 8  rs11218343       -0.2697      0.0410                      C
## 9  rs10498633       -0.1044      0.0199                      T
## 10  rs8093731       -0.6136      0.1123                      T
## 11  rs4147929        0.1348      0.0224                      A
## 12  rs3865444       -0.0954      0.0175                      A
##    other_allele.exposure eaf.exposure pval.exposure     gene.exposure
## 1                      G        0.197      7.73e-15               CR1
## 2                      C        0.409      1.66e-26              BIN1
## 3                      A        0.266      3.05e-08             CD2AP
## 4                      A        0.276      1.60e-08 HLA-DRB5-HLA-DRB1
## 5                      G        0.338      8.76e-10             EPHA1
## 6                      T        0.366      3.27e-09             PTK2B
## 7                      T        0.379      9.63e-17               CLU
## 8                      T        0.039      4.98e-11             SORL1
## 9                      G        0.217      1.47e-07      SLC24A4-RIN3
## 10                     C        0.017      4.63e-08              DSG2
## 11                     G        0.190      1.70e-09             ABCA7
## 12                     C        0.307      5.12e-08              CD33
##    samplesize.exposure exposure mr_keep.exposure pval_origin.exposure
## 1                54162       AD             TRUE             reported
## 2                54162       AD             TRUE             reported
## 3                54162       AD             TRUE             reported
## 4                54162       AD             TRUE             reported
## 5                54162       AD             TRUE             reported
## 6                54162       AD             TRUE             reported
## 7                54162       AD             TRUE             reported
## 8                54162       AD             TRUE             reported
## 9                54162       AD             TRUE             reported
## 10               54162       AD             TRUE             reported
## 11               54162       AD             TRUE             reported
## 12               54162       AD             TRUE             reported
##    id.exposure data_source.exposure
## 1       5Hjhu1             textfile
## 2       5Hjhu1             textfile
## 3       5Hjhu1             textfile
## 4       5Hjhu1             textfile
## 5       5Hjhu1             textfile
## 6       5Hjhu1             textfile
## 7       5Hjhu1             textfile
## 8       5Hjhu1             textfile
## 9       5Hjhu1             textfile
## 10      5Hjhu1             textfile
## 11      5Hjhu1             textfile
## 12      5Hjhu1             textfile
```

```
### read outcome data (12 SNPs from any stroke GWAS)
any_stroke_outcome_dat <- read_outcome_data(snps = AD_exposure_dat$SNP,
                                    filename = "C:/Users/12601/Desktop/MR_modifition/TwoSampleM
R_outcome AD and AS.csv",
                                    sep = ",",snp_col = "SNP",beta_col = "beta",se_col = "se",
                                    effect_allele_col = "effect_allele",other_allele_col = "oth
er_allele",
                                    gene_col = "gene",samplesize_col = "samplesize")
###print outcome data
any_stroke_outcome_dat
```

```
##           SNP beta.outcome se.outcome effect_allele.outcome
## 1   rs6656401       0.0075     0.0116                     A
## 2   rs6733839      -0.0075     0.0087                     T
## 3  rs10948363       0.0034     0.0093                     G
## 4   rs9271192       0.0006     0.0104                     C
## 5  rs11771145       0.0152     0.0082                     A
## 6  rs28834970      -0.0014     0.0083                     C
## 7   rs9331896       0.0002     0.0083                     C
## 8  rs11218343       0.0060     0.0149                     C
## 9  rs10498633       0.0094     0.0102                     T
## 10  rs8093731       0.0079     0.0237                     T
## 11  rs4147929       0.0160     0.0103                     A
## 12  rs3865444      -0.0133     0.0089                     A
##    other_allele.outcome eaf.outcome pval.outcome      gene.outcome
## 1                     G      0.1740      0.51990               CR1
## 2                     C      0.3832      0.38910              BIN1
## 3                     A      0.2423      0.71650             CD2AP
## 4                     A      0.2759      0.95380 HLA-DRB5-HLA-DRB1
## 5                     G      0.3997      0.06316             EPHA1
## 6                     T      0.3437      0.86590             PTK2B
## 7                     T      0.3874      0.98430               CLU
## 8                     T      0.2166      0.68740             SORL1
## 9                     G      0.2063      0.35630      SLC24A4-RIN3
## 10                    C      0.1366      0.73820              DSG2
## 11                    G      0.2288      0.12010             ABCA7
## 12                    C      0.2945      0.13630              CD33
##    samplesize.outcome    outcome mr_keep.outcome pval_origin.outcome id.outcome
## 1              521612 any_stroke            TRUE            reported     IUuduP
## 2              521612 any_stroke            TRUE            reported     IUuduP
## 3              521612 any_stroke            TRUE            reported     IUuduP
## 4              521612 any_stroke            TRUE            reported     IUuduP
## 5              521612 any_stroke            TRUE            reported     IUuduP
## 6              521612 any_stroke            TRUE            reported     IUuduP
## 7              521612 any_stroke            TRUE            reported     IUuduP
## 8              521612 any_stroke            TRUE            reported     IUuduP
## 9              521612 any_stroke            TRUE            reported     IUuduP
## 10             521612 any_stroke            TRUE            reported     IUuduP
## 11             521612 any_stroke            TRUE            reported     IUuduP
## 12             521612 any_stroke            TRUE            reported     IUuduP
##    data_source.outcome
## 1             textfile
## 2             textfile
## 3             textfile
## 4             textfile
## 5             textfile
## 6             textfile
## 7             textfile
## 8             textfile
## 9             textfile
## 10            textfile
## 11            textfile
## 12            textfile
```

```
### harmonise exposure data and outcome data
dat <- harmonise_data(AD_exposure_dat, any_stroke_outcome_dat)
```

```
## Harmonising AD (5Hjhu1) and any_stroke (IUuduP)
```

```
### set up unit for the exposure
dat$units.exposure <- "OR"

### set up unit for the outcome
dat$units.outcome <- "OR"
class(dat)
```

```
## [1] "data.frame"
```

```
### run Steiger filtering for each SNP
dat2 <- steiger_filtering(dat)
```

```
## Estimating correlation for quantitative trait.
```

```
## This method is an approximation, and may be numerically unstable.
```

```
## Ideally you should estimate r directly from independent replication samples.
```

```
## Use get_r_from_lor for binary traits.
```

```
## Estimating correlation for quantitative trait.
```

```
## This method is an approximation, and may be numerically unstable.
```

```
## Ideally you should estimate r directly from independent replication samples.
```

```
## Use get_r_from_lor for binary traits.
```

```
### MR analysis excluding instruments with the wrong direction of effects
mr_results <- mr(subset(dat2,steiger_dir))
```

```
## Analysing '5Hjhu1' on 'IUuduP'
```

```
### print mr_results
mr_results
```

```
##   id.exposure id.outcome    outcome exposure                    method nsnp
## 1      5Hjhu1     IUuduP any_stroke       AD                  MR Egger   12
## 2      5Hjhu1     IUuduP any_stroke       AD           Weighted median   12
## 3      5Hjhu1     IUuduP any_stroke       AD Inverse variance weighted   12
## 4      5Hjhu1     IUuduP any_stroke       AD               Simple mode   12
## 5      5Hjhu1     IUuduP any_stroke       AD             Weighted mode   12
##              b         se      pval
## 1 -0.019046666 0.04060751 0.6490977
## 2 -0.013306328 0.02535704 0.5997509
## 3 -0.007022153 0.01874067 0.7078827
## 4 -0.007729140 0.03539387 0.8311354
## 5 -0.016001141 0.02875307 0.5890098
```
