# Supplementary material for: Stroke and Alzheimer’s Disease: A Mendelian Randomization Study
Source: Front Genet. 2020 Jul 14;11:581. doi: 10.3389/fgene.2020.00581 (PMC7371994; doi:10.3389/fgene.2020.00581)
Supplement: Supplementary file 8 [file Table_3.docx]

| Supplementary Table 3: P values for 11 genetic variants with nine potential risk factors for dementia | | | | |
| --- | --- | --- | --- | --- |
| SNP | Traits | PMID | Sample size | P value |
| rs880315 | Years of educational attainment | 27225129 | 328917 | 0.111 |
| rs12037987 | Years of educational attainment | 27225129 | 328917 | 0.327 |
| rs16896398 | Age completed full time education | UKBB | 226899 | 0.0759 |
| rs7859727 | NA | NA | NA | NA |
| rs2295786 | NA | NA | NA | NA |
| rs35436 | Years of educational attainment in males | 27225129 | 147474 | 0.254 |
| rs9526212 | Years of educational attainment | 27225129 | 328917 | 0.134 |
| rs8103309 | Years of educational attainment in females | 27225129 | 181443 | 0.147 |
| rs1052053 | NA | NA | NA | NA |
| rs4959130 | NA | NA | NA | NA |
| rs12445022 | Years of educational attainment | 23722424 | 101069 | 0.251 |
| rs880315 | Hearing difficulty or problems | UKBB | 323978 | 0.213 |
| rs12037987 | Hearing difficulty or problems with background noise | UKBB | 330759 | 0.122 |
| rs16896398 | Hearing difficulty or problems with background noise | UKBB | 330759 | 2.18E-08 |
| rs7859727 | Hearing aid user | UKBB | 204240 | 0.172 |
| rs2295786 | Hearing difficulty or problems | UKBB | 323978 | 5.32E-02 |
| rs35436 | Hearing difficulty or problems | UKBB | 323978 | 0.292 |
| rs9526212 | Hearing difficulty or problems | UKBB | 323978 | 0.153 |
| rs8103309 | Hearing difficulty or problems with background noise | UKBB | 330759 | 5.83E-02 |
| rs1052053 | NA | NA | NA | NA |
| rs4959130 | Hearing aid user | UKBB | 204240 | 8.81E-03 |
| rs12445022 | Hearing difficulty or problems | UKBB | 323978 | 0.246 |
| rs880315 | Number of days or week of vigorous activity 10+ minutes | UKBB | 321533 | 0.103 |
| rs12037987 | Number of days or week of vigorous physical activity 10+ minutes | UKBB | 321533 | 0.123 |
| rs16896398 | Number of days or week of vigorous physical activity 10+ minutes | UKBB | 321533 | 1.98E-04 |
| rs7859727 | Number of days or week of vigorous physical activity 10+ minutes | UKBB | 321533 | 0.167 |
| rs2295786 | Number of days or week of moderate physical activity 10+ minutes | UKBB | 321309 | 0.216 |
| rs35436 | Types of physical activity in last 4 weeks: strenuous sports | UKBB | 335599 | 0.35 |
| rs9526212 | Number of days or week of vigorous physical activity 10+ minutes | UKBB | 321533 | 0.116 |
| rs8103309 | Duration of vigorous activity | UKBB | 183731 | 0.156 |
| rs1052053 | NA | NA | NA | NA |
| rs4959130 | Number of days or week of vigorous physical activity 10+ minutes | UKBB | 321533 | 0.0654 |
| rs12445022 | Number of days or week of moderate physical activity 10+ minutes | UKBB | 321309 | 0.0608 |
| rs880315 | hypertension | 27618447 | 183273 | 8.41E-07 |
| rs12037987 | Self-reported essential hypertension | UKBB | 337159 | 0.347 |
| rs16896398 | Self-reported essential hypertension | UKBB | 337159 | 0.0799 |
| rs7859727 | Self-reported hypertension | UKBB | 337159 | 0.2 |
| rs2295786 | Self-reported hypertension | UKBB | 337159 | 0.244 |
| rs35436 | Self-reported essential hypertension | UKBB | 337159 | 0.0298 |
| rs9526212 | Self-reported hypertension | UKBB | 337159 | 0.0253 |
| rs8103309 | Self-reported essential hypertension | UKBB | 337159 | 0.186 |
| rs1052053 | NA | NA | NA | NA |
| rs4959130 | Self-reported hypertension | UKBB | 337159 | 0.0263 |
| rs12445022 | Self-reported hypertension | UKBB | 337159 | 0.0328 |
| rs880315 | hypertension | 27618447 | 183273 | 8.41E-07 |
| rs12037987 | Self-reported essential hypertension | UKBB | 337159 | 0.347 |
| rs16896398 | Self-reported essential hypertension | UKBB | 337159 | 0.0799 |
| rs7859727 | Self-reported hypertension | UKBB | 337159 | 0.2 |
| rs2295786 | Self-reported hypertension | UKBB | 337159 | 0.244 |
| rs35436 | Self-reported essential hypertension | UKBB | 337159 | 0.0298 |
| rs9526212 | Self-reported hypertension | UKBB | 337159 | 0.0253 |
| rs8103309 | Self-reported essential hypertension | UKBB | 337159 | 0.186 |
| rs1052053 | NA | NA | NA | NA |
| rs4959130 | Self-reported hypertension | UKBB | 337159 | 0.0263 |
| rs12445022 | Self-reported hypertension | UKBB | 337159 | 0.0328 |
| rs880315 | NA | NA | NA | NA |
| rs12037987 | NA | NA | NA | NA |
| rs16896398 | Type II diabetes | 28566273 | 159208 | 7.80E-04 |
| rs7859727 | Type II diabetes | 28566273 | 159208 | 2.50E-03 |
| rs2295786 | Type II diabetes | 28566273 | 159208 | 0.28 |
| rs35436 | Type II diabetes adjusted for BMI | 28566273 | 159208 | 0.3 |
| rs9526212 | Type II diabetes | 28566273 | 159208 | 2.80E-03 |
| rs8103309 | NA | NA | NA | NA |
| rs1052053 | NA | NA | NA | NA |
| rs4959130 | Type II diabetes | 28566273 | 159208 | 0.12 |
| rs12445022 | Type II diabetes | UKBB | 110452 | 0.0328 |
| rs880315 | Body mass index | 25673413 | 322154 | 0.256 |
| rs12037987 | Body mass index | 28892062 | 173430 | 0.0464 |
| rs16896398 | Body mass index | 25673413 | 322154 | 0.295 |
| rs7859727 | Body mass index | UKBB | 336107 | 0.0921 |
| rs2295786 | Body mass index | UKBB | 336107 | 0.0168 |
| rs35436 | Body mass index | UKBB | 336107 | 0.126 |
| rs9526212 | Body mass index | UKBB | 336107 | 0.117 |
| rs8103309 | Body mass index | UKBB | 336107 | 1.07 E-04 |
| rs1052053 | NA | NA | NA | NA |
| rs4959130 | NA | NA | NA | NA |
| rs12445022 | Body mass index | 28892062 | 173430 | 0.113 |
| rs880315 | Cigarettes per day | 20418890 | 38181 | 0.151 |
| rs12037987 | Smoked cigarette or pipe within last hour | UKBB | 30270 | 0.209 |
| rs16896398 | Smoking or smokers in household | UKBB | 311142 | 0.218 |
| rs7859727 | Past tobacco smoking | UKBB | 310749 | 0.077 |
| rs2295786 | Cigarettes per day | 20418890 | 38181 | 0.0256 |
| rs35436 | Number of cigarettes previously smoked daily | UKBB | 78291 | 0.0759 |
| rs9526212 | Smoking or smokers in household | UKBB | 311142 | 0.336 |
| rs8103309 | Smoking status: previous | UKBB | 336024 | 0.0595 |
| rs1052053 | NA | NA | NA | NA |
| rs4959130 | Smoking or smokers in household | UKBB | 311142 | 0.231 |
| rs12445022 | Smoking status: previous | 28892062 | 336024 | 0.0814 |
| rs880315 | Self-reported depression | UKBB | 337159 | 0.19 |
| rs12037987 | Recurrent depressive disorder | UKBB | 337199 | 0.142 |
| rs16896398 | Recurrent depressive disorder | UKBB | 337199 | 0.13 |
| rs7859727 | Depressive symptoms | 27089181 | 180866 | 0.0785 |
| rs2295786 | Self-reported depression | UKBB | 337159 | 0.0759 |
| rs35436 | Self-reported post-natal depression | UKBB | 337159 | 0.123 |
| rs9526212 | Longest period of depression | UKBB | 46884 | 0.19 |
| rs8103309 | Seen a psychiatrist for nerves, anxiety, tension or depression | UKBB | 335888 | 0.0381 |
| rs1052053 | NA | NA | NA | NA |
| rs4959130 | Self-reported depression | UKBB | 337159 | 0.0806 |
| rs12445022 | Self-reported depression | UKBB | 337159 | 0.141 |
| rs880315 | NA | NA | NA | NA |
| rs12037987 | NA | NA | NA | NA |
| rs16896398 | NA | NA | NA | NA |
| rs7859727 | NA | NA | NA | NA |
| rs2295786 | NA | NA | NA | NA |
| rs35436 | NA | NA | NA | NA |
| rs9526212 | Loneliness, isolation | UKBB | 332263 | 0.0282 |
| rs8103309 | NA | NA | NA | NA |
| rs1052053 | NA | NA | NA | NA |
| rs4959130 | Loneliness, isolation | UKBB | 332263 | 0.0585 |
| rs12445022 | NA | NA | NA | NA |
