# Supplementary material for: Stroke and Alzheimer’s Disease: A Mendelian Randomization Study
Source: Front Genet. 2020 Jul 14;11:581. doi: 10.3389/fgene.2020.00581 (PMC7371994; doi:10.3389/fgene.2020.00581)
Supplement: Supplementary file 9 [file Table_4.docx]

| Supplementary Table **4**: P values for 11 genetic variants with risk factors for dementia | | | | |
| --- | --- | --- | --- | --- |
| SNP | Traits | PMID | Sample size | P value |
| rs880315 | Telomere length | NA | NA | NA |
| rs12037987 | Telomere length | NA | NA | NA |
| rs16896398 | Telomere length | NA | NA | NA |
| rs7859727 | Telomere length | NA | NA | NA |
| rs2295786 | Telomere length | NA | NA | NA |
| rs35436 | Telomere length | NA | NA | NA |
| rs9526212 | Telomere length | NA | NA | NA |
| rs8103309 | Telomere length | NA | NA | NA |
| rs1052053 | Telomere length | NA | NA | NA |
| rs4959130 | Telomere length | NA | NA | NA |
| rs12445022 | Telomere length | NA | NA | NA |
| rs880315 | Tobacco smoking: occasionally | UKBB | 83133 | 0.243 |
| rs12037987 | Ever stopped smoking for 6+ months | UKBB | 81538 | 0.264 |
| rs16896398 | Past tobacco smoking | UKBB | 310749 | 0.127 |
| rs7859727 | Past tobacco smoking | UKBB | 310749 | 0.077 |
| rs2295786 | Tobacco smoking: smokes on most or all days | UKBB | 83133 | 0.084 |
| rs35436 | Ever stopped smoking for 6+ months | UKBB | 81538 | 0.170 |
| rs9526212 | Smoking status: current | UKBB | 336024 | 0.247 |
| rs8103309 | Past tobacco smoking | UKBB | 310749 | 0.112 |
| rs1052053 | Past tobacco smoking | NA | NA | NA |
| rs4959130 | Tobacco smoking: smokes on most or all days | UKBB | 83133 | 0.356 |
| rs12445022 | Past tobacco smoking | UKBB | 310749 | 0.0856 |
| rs880315 | Treatment with vitamin d product | UKBB | 337159 | 0.0827 |
| rs12037987 | Treatment with vitamin d product | UKBB | 337159 | 0.0655 |
| rs16896398 | Treatment with calcium+vitamin d 500units tablet | UKBB | 337159 | 0.128 |
| rs7859727 | Treatment with vitamin d product | NA | NA | NA |
| rs2295786 | Treatment with vitamin a+d capsule | UKBB | 337159 | 0.215 |
| rs35436 | Vitamin and mineral supplements: vitamin D | UKBB | 335591 | 0.251 |
| rs9526212 | Treatment with calcium+vitamin d 500units tablet | UKBB | 337159 | 0.039 |
| rs8103309 | Treatment with vitamin d product | UKBB | 337159 | 0.306 |
| rs1052053 | Treatment with vitamin d product | NA | NA | NA |
| rs4959130 | Treatment with vitamin a+d capsule | UKBB | 337159 | 0.0455 |
| rs12445022 | Treatment with calcium+vitamin d 500units tablet | UKBB | 337159 | 0.0689 |
| rs880315 | homocysteine | NA | NA | NA |
| rs12037987 | homocysteine | NA | NA | NA |
| rs16896398 | homocysteine | NA | NA | NA |
| rs7859727 | homocysteine | NA | NA | NA |
| rs2295786 | homocysteine | NA | NA | NA |
| rs35436 | homocysteine | NA | NA | NA |
| rs9526212 | homocysteine | NA | NA | NA |
| rs8103309 | homocysteine | NA | NA | NA |
| rs1052053 | homocysteine | NA | NA | NA |
| rs4959130 | homocysteine | NA | NA | NA |
| rs12445022 | homocysteine | NA | NA | NA |
| rs880315 | systolic blood pressure | UKBB | 317754 | 5.62E-14 |
| rs12037987 | systolic blood pressure | UKBB | 317754 | 2.72E-13 |
| rs16896398 | systolic blood pressure | 21909115 | 69395 | 2.89E-05 |
| rs7859727 | systolic blood pressure | NA | NA | NA |
| rs2295786 | systolic blood pressure | 21909115 | 69395 | 0.0387 |
| rs35436 | systolic blood pressure | UKBB | 317754 | 5.10E-13 |
| rs9526212 | systolic blood pressure | UKBB | 317754 | 7.19E-04 |
| rs8103309 | systolic blood pressure | UKBB | 317754 | 0.155 |
| rs1052053 | systolic blood pressure | NA | NA | NA |
| rs4959130 | systolic blood pressure | UKBB | 317754 | 0.220 |
| rs12445022 | systolic blood pressure | 21909115 | 69395 | 0.343 |
| rs880315 | 2 hour fasting glucose | 22885924 | 42854 | 0.0111 |
| rs12037987 | 2 hour fasting glucose | NA | NA | NA |
| rs16896398 | 2 hour fasting glucose | 20081857 | 15234 | 0.0287 |
| rs7859727 | 2 hour fasting glucose | 20081857 | 15234 | 0.0771 |
| rs2295786 | 2 hour fasting glucose | NA | NA | NA |
| rs35436 | 2 hour fasting glucose | NA | NA | NA |
| rs9526212 | 2 hour fasting glucose | NA | NA | NA |
| rs8103309 | 2 hour fasting glucose | NA | NA | NA |
| rs1052053 | 2 hour fasting glucose | NA | NA | NA |
| rs4959130 | 2 hour fasting glucose | NA | NA | NA |
| rs12445022 | 2 hour fasting glucose | NA | NA | NA |
| rs880315 | Medication for cholesterol, blood pressure or diabetes: insulin | UKBB | 154702 | 0.113 |
| rs12037987 | Started insulin within one year diagnosis of diabetes | UKBB | 15397 | 0.0781 |
| rs16896398 | Treatment with insulin product | UKBB | 337159 | 0.254 |
| rs7859727 | Treatment with insulin product | UKBB | 337159 | 0.0443 |
| rs2295786 | Treatment with insulin product | UKBB | 337159 | 0.107 |
| rs35436 | Treatment with insulin product | insulin | insulin | insulin |
| rs9526212 | Started insulin within one year diagnosis of diabetes | UKBB | 15397 | 0.0195 |
| rs8103309 | Medication for cholesterol, blood pressure or diabetes: insulin | UKBB | 154702 | 0.219 |
| rs1052053 | Medication for cholesterol, blood pressure or diabetes: insulin | NA | NA | NA |
| rs4959130 | Medication for cholesterol, blood pressure or diabetes: insulin | UKBB | 154702 | 0.332 |
| rs12445022 | Insulin-like growth factor 1 | 28887542 | 9961 | 0.0907 |
| rs880315 | High density lipoprotein | 24097068 | 187167 | 0.0866 |
| rs12037987 | High density lipoprotein | NA | NA | NA |
| rs16896398 | High density lipoprotein | NA | NA | NA |
| rs7859727 | High density lipoprotein | 20686565 | 99900 | 0.16 |
| rs2295786 | High density lipoprotein | NA | NA | NA |
| rs35436 | High density lipoprotein | NA | NA | NA |
| rs9526212 | High density lipoprotein | NA | NA | NA |
| rs8103309 | High density lipoprotein | NA | NA | NA |
| rs1052053 | High density lipoprotein | NA | NA | NA |
| rs4959130 | High density lipoprotein | NA | NA | NA |
| rs12445022 | High density lipoprotein | 28887542 | 9961 | 0.0898 |
