# Supplementary material for: Stroke and Alzheimer’s Disease: A Mendelian Randomization Study
Source: Front Genet. 2020 Jul 14;11:581. doi: 10.3389/fgene.2020.00581 (PMC7371994; doi:10.3389/fgene.2020.00581)
Supplement: Supplementary file 10 [file Table_5.DOCX]

**Supplementary Table 5**. Characteristics of fourteen genetic variants in AD and any stroke datasets

| SNP | Chr | Nearby Genes | EA^a^ | NEA | EAF^b^ | AD GWAS | | | Any stroke GWAS | | |
| --- | --- | --- | --- | --- | --- | --- | --- | --- | --- | --- | --- |
|  |  |  |  |  |  | Beta^c^ | SE^c^ | *P* value^c^ | Beta^d^ | SE^d^ | *P* value^d^ |
| rs6656401 | 1 | *CR1* | A | G | 0.197 | 0.1567 | 0.0202 | 7.73E-15 | 0.0075 | 0.0116 | 0.5199 |
| rs6733839 | 2 | *BIN1* | T | C | 0.409 | 0.188 | 0.0176 | 1.66E-26 | -0.0075 | 0.0087 | 0.3891 |
| rs10948363 | 6 | *CD2AP* | G | A | 0.266 | 0.0978 | 0.0177 | 3.05E-08 | 0.0034 | 0.0093 | 0.7165 |
| rs9271192 | 6 | *HLA-DRB5–*  *HLA-DRB1* | C | A | 0.276 | 0.1044 | 0.0206 | 1.60E-08 | 0.0006^p^ | 0.0104^p^ | 0.9538^p^ |
| rs11771145 | 7 | *EPHA1* | A | G | 0.338 | -0.1024 | 0.0167 | 8.76E-10 | 0.0152 | 0.0082 | 0.06316 |
| rs28834970 | 8 | *PTK2B* | C | T | 0.366 | 0.0959 | 0.0162 | 3.27E-09 | -0.0014 | 0.0083 | 0.8659 |
| rs9331896 | 8 | *CLU* | C | T | 0.379 | -0.1457 | 0.0175 | 9.63E-17 | 0.0002 | 0.0083 | 0.9843 |
| rs983392 | 11 | *MS4A6A* | G | A | 0.403 | -0.1084 | 0.0163 | 2.76E-11 | 0.0198 | 0.0089 | 0.02593 |
| rs10792832 | 11 | *PICALM* | A | G | 0.358 | -0.1297 | 0.0161 | 6.53E-16 | -0.0173 | 0.0082 | 0.03544 |
| rs11218343 | 11 | *SORL1* | C | T | 0.039 | -0.2697 | 0.041 | 4.98E-11 | 0.006 | 0.0149 | 0.6874 |
| rs10498633 | 14 | *SLC24A4-*  *RIN3* | T | G | 0.217 | -0.1044 | 0.0199 | 1.47E-07 | 0.0094 | 0.0102 | 0.3563 |
| rs8093731 | 18 | *DSG2* | T | C | 0.017 | -0.6136 | 0.1123 | 4.63E-08 | 0.0079 | 0.0237 | 0.7382 |
| rs4147929 | 19 | *ABCA7* | A | G | 0.190 | 0.1348 | 0.0224 | 1.70E-09 | 0.016 | 0.0103 | 0.1201 |
| rs3865444 | 19 | *CD33* | A | C | 0.307 | -0.0954 | 0.0175 | 5.12E-08 | -0.0133 | 0.0089 | 0.1363 |

Abbreviation: SNP, single-nucleotide polymorphism; Chr, chromosome; EA, effect allele; NEA, non-effect allele; EAF, effect allele frequency; AD, Alzheimer’s disease; GWAS, genome-wide association studies; SE, standard error.

a: effect allele associated with AD.

b: the frequency of effect allele associated with AD.

c: the summary statistics for SNP, including Beta, standard error and *P* value, were obtained from AD GWAS dataset. Beta > 0 indicates this effect allele increases the AD risk. Otherwise, it reduces the AD risk (Beta < 0).

d: the summary statistics for SNP, including Beta, standard error and *P* value, were obtained from any stroke dataset. The Beta was obtained based on the effect allele associated AD. Beta > 0 indicates this effect allele increases the stroke risk. Otherwise, it reduces the stroke risk (Beta < 0).

p: summary statistics for proxy SNP rs9271162 of rs9271192 (r^2^= 0.96), and the effect allele and reference allele of rs9271162 is G and T, respectively.
